# Unraveling the Role of the Tyrosine Tetrad from the Binding Site of the Epigenetic Writer MLL3 in the Catalytic Mechanism and Methylation Multiplicity

**DOI:** 10.3390/ijms231810339

**Published:** 2022-09-07

**Authors:** Kevin Blanco-Esperguez, Iñaki Tuñón, Johannes Kästner, Fernando Mendizábal, Sebastián Miranda-Rojas

**Affiliations:** 1 Departamento de Ciencias Químicas, Facultad de Ciencias Exactas, Universidad Andres Bello, República 275, Santiago 8370146, Chile; 2 Departamento de Química Física, Universidad de Valencia, 46100 Burjasot, Spain; 3 Institut für Theoretische Chemie, Universität Stuttgart, Pfaffenwaldring 55, 70569 Stuttgart, Germany; 4 Departamento de Química, Facultad de Ciencias, Universidad de Chile, P.O. Box 653, Las Palmeras 3425, Ñuñoa, Santiago 7800003, Chile

**Keywords:** cancer, epigenetics, mutants, enzyme catalysis, product specificity, QM/MM

## Abstract

MLL3, also known as KMT2C, is a lysine mono-methyltransferase in charge of the writing of an epigenetic mark on lysine 4 from histone 3. The catalytic site of MLL3 is composed of four tyrosines, namely, Y44, Y69, Y128, and Y130. Tyrosine residues are highly conserved among lysine methyltransferases’ catalytic sites, although their complete function is still unclear. The exploration of how modifications on these residues from the enzymatic machinery impact the enzymatic activity of MLL3 could shed light transversally into the inner functioning of enzymes with similar characteristics. Through the use of QMMM calculations, we focus on the effect of the mutation of each tyrosine from the catalytic site on the enzymatic activity and the product specificity in the current study. While we found that the mutations of Y44 and Y128 by phenylalanine inactivated the enzyme, the mutation of Y128 by alanine reactivated the enzymatic activity of MLL3. Moreover, according to our models, the Y128A mutant was even found to be capable of di- and tri-methylate lysine 4 from histone 3, what would represent a gain of function mutation, and could be responsible for the development of diseases. Finally, we were able to establish the inactivation mechanism, which involved the use of Y130 as a water occlusion structure, whose conformation, once perturbed by its mutation or Y128 mutant, allows the access of water molecules that sequester the electron pair from lysine 4 avoiding its methylation process and, thus, increasing the barrier height.

## 1. Introduction

In recent decades, the histone methylation process has become a topic of increasing interest because of its implication in the gene-regulation process [1], as it is linked to the chromatin-folding state, thus regulating the accessibility of transcription factors towards DNA. This process, also known as writing an epigenetic mark, occurs in sites located at the side chain of basic residues, such as lysine (K) located at the N-terminal domain of histones, process catalyzed by lysine methyltransferases (KMTs). In this context, histone methylation has been involved in gene activation and gene silencing, depending on the target lysine from the N-terminal domain [2]. The source of the methyl group used by methyltransferases comes from the S-adenosyl methionine (SAM) cofactor that acts as the methyl donor during the mechanism [3]. Chemically, the lysine can be mono-, di-, or tri-methylated at the ε-nitrogen from the side chain, namely, Nε(K), which is designated as a target for methylation.

Despite the fact that the methylation process would only involve minor conformational changes in the main structure of the N-terminal domain, the amount of information codified through this mechanism is highly versatile in terms of its role in activating and silencing. In fact, it was proposed that the role of di-methylated H3K4 is related to determining a permissive chromatin environment, while the tri-methylated state corresponds to a fully active chromatin conformation [4]. At the histone N-terminal domain, there is a large variety of lysine residues that are methylated in humans, among which in histone 3 (H3) it is possible to find, in addition to H3K4, lysine 9 (H3K9), lysine 27 (H3K27), lysine 36 (H3K36), and lysine 79 (H3K79) [5]; whereas in histone 4 (H4), the most common is lysine 20 (H4K20) [6]. These lysines and their methylation processes are somehow involved in chromatin regulation and DNA-expression functions [7]. The high number of sites that can be methylated together with methylation multiplicity illustrates the complexity of this regulation mechanism and also points out how its deregulation may induce the origin of several diseases [7,8]. The dysregulation in the methyltransferases’ activity is a common cause of genetic disorders [9], cancer disease [10], and metabolic and degenerative disorders [8], all somehow related to alterations in the epigenetic regulation pathways. Recent studies have shown that alterations in the epigenetic regulation pathways are commonly related to specific mutations in some methyltransferase enzymes, which has been identified in several cancer lines along with other diseases [11].

**Scheme 1:**
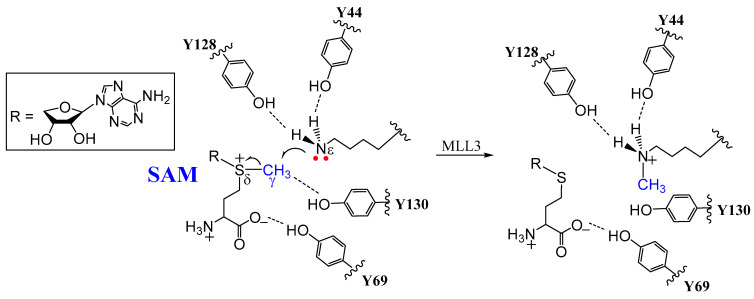
Chemical representation of the catalytic machinery of MLL3 and its involvement in the reaction mechanism for the methyl-transfer process.

Among the KMTs, the mixed-lineage-leukemia MLL family of enzymes are found in humans and bony vertebrates in general. In particular, MLL3 regulates the expression of a tumor-suppressor gene, whose malfunctioning has been related to several types of cancer disease. For example, MLL3 is a haploinsufficient 7q tumor suppressor in acute myeloid leukemia [12] and its inactivation in mice leads to the formation of ureter epithelial tumors [13]. The decrease in the expression of MLL3 has been observed in several types of breast cancer [14] and is correlated with low survival rates in patients with gastric cancer [15]. The MLL3 protein subunit by itself is catalytically inactive, and the binding of RBPP5 and ASH2L protein subunits are the responsible of activating catalytic machinery, forming a trimeric complex, hereafter named as M3RA [16,17]. The enzymatic reaction for the methyl-transfer process is a two-stage mechanism that begins with the deprotonation of the lysine residue, followed by the methyl transfer from the SAM cofactor to the target lysine, a step presented in Scheme 1. The active site of the MLL3 subunit contains a tetrad of tyrosine residues (Y44, Y69, Y128, Y130) that have a crucial role in the process of the deprotonation and methylation of the target lysine and also in the binding of the cofactor [18]. The correlation between the residues’ numbering used in this article and the one used in the article by Li et al. [19], associated with the full-length protein, is detailed in Table S1 in the Supplementary Materials. MLL3 is a mono-methyltransferase, meaning it can transfer only one methyl group to the target lysine. The visual inspection of the crystal structure with PDB code 5F6K [19] shows that the presence of both Y44 and Y128 in MLL3 entails a steric impediment to accommodate the first methyl group in such way that allows the rotation and attack of a new methyl group. This has also been proposed for other KMT enzymes, such as SET7/9, in which the hydroxyl group from Y305, which would be analogous to Y128 in MLL3, could be responsible for steric hindrance limiting the methylation [20]. The sequence analysis of KMTs, including MLL enzymes, suggests that other KMTs can di- or tri-methylate because only two of the four tyrosine residues observed in MLL3 are fully conserved, one involved in the lysine binding (Y44) and the other in the structure of the oxy-anion ring (Y130) [21]. However, there are cases, such as DIM-5, a sub-type of the SUV39H1 KMT enzymes, in which when a third tyrosine analogous to Y44 from MLL3 (Y178 from DIM-5) is mutated to valine, it completely abolishes its enzymatic function [20]. On the other hand, it has been proposed that the fact that in some KMTs, such as G9a, the presence of a proline residue, where in other enzymes it would be located a tyrosine instead, provides the enzyme with the ability to di-methylate lysine [18]. This is probably because of an increase in the size of the cavity necessary to accommodate a mono-methylated lysine substrate. In SET7/9, a mono-methyltransferase, such as MLL3, the Y245A mutant tyrosine that is analogous to Y44 in MLL3, led to a reduction in the enzymatic activity towards mono-methylation, although it was possible to use mono- and di-methylated substrates to form di- and tri-methylated lysine products [18,20]. In a similar context, the Y305F mutant (Y128 in MLL3) resulted in an increase in the activity of SET7/9 towards di-methylation, and it even presented traces of tri-methylated product [20]. Similar to SET7/9, we would expect that similar mutations of analogous residues in MLL3 may lead to a change in its product specificity, although it is not possible to discard the idea that similar mutations could abolish its activity as it has been observed with other enzymes. The MLL3 enzyme itself has been found to be highly affected by mutations in many cancer lines [12]. In this context, the mutant Y128C obtained from MLL3 found in large intestine cancer presented a change in the product specificity producing an aberrant trimethylation of the target lysine [22]. Genome sequencing has allowed us to identify several somatic mutations in MLL3 found in many diseases, such as non-Hodgkin’s lymphoma [23], bladder cancer [24], breast cancer [14], medulloblastoma [25], acute myeloid leukemia [26], and epithelial lymphoma T cells [27]. In this regard, some mutations in MLL enzymes frequently observed in glioblastoma, pancreatic cancer, melanoma, and breast cancer [28] have been identified.

In line with the background detailed above, we explore the role of the tetrad of tyrosines in the catalytic site of MLL3 by mutating these residues to determine their role in catalytic efficiency and predict their impact on the product specificity towards the methyl-transfer process. Our results shed light on the effect of such mutations on the generation of impaired or gain-of-function states of this enzyme. Considering that MLL3 is directly involved in the regulation mechanism of gene transcription, the findings related to this study allow us to set part of the molecular basis for the etiology of certain diseases, and consequently make this enzyme an attractive target for drug design in its different pathological states.

## 2. Results and Discussion

The present work focused on studying the role of tyrosine tetrad obtained from the catalytic site of M3RA, namely, Y44, Y128, Y69, and Y130. We aimed to study the changes in the barrier height (∆E^≠^) of the methyl-transfer process derived from the mutation of these four residues. While, Y44 and Y128 were mutated by phenylalanine and alanine, Y69 and Y130 were mutated only by phenylalanine. This was performed to unveil the influence of each residue on the energy barrier in the methyl-transfer process. Finally, we proceeded to study the effect of each mutation on product specificity.

### 2.1. Monomethyl-Transfer Reaction: Y44F/Y44A and Y128F/Y128A Mutants

According to the crystal structure used in this study, the main role of these two residues corresponded to the proper anchoring of the H3K4 substrate lysine, where the oxygens from the hydroxyl groups of Y44 and Y128 pointed towards Nε(K4) obtained from the lysine. However, as described above, there were KMT enzymes capable of performing the methyl-transfer reaction with just one tyrosine residue in these positions, which raised the question of how necessary the presence of both Y44 and Y128 for the catalytic efficiency was, and which one, if any, was particularly essential for the process. To determine the role of the mutations of Y44 and Y128 in the catalytic efficiency, we systematically proceeded by mutating the two residues by phenylalanine and alanine to define the role of the hydroxyl group and the aromatic ring from the tyrosines in the anchoring process of the lysine substrate and its effect on the reaction process. In all cases, we proceeded by selecting twenty-five conformations obtained from the MD simulations of the respective mutant with deprotonated K4, assuming that the deprotonation process had already occurred. In this way, we focused our analysis on the effect of the mutation on the methylation process, the rate-limiting step. In any case, a detailed explanation of the arguments from which we assumed that the deprotonation process would also occur in the mutants was presented in Section S1 of the Supplementary Materials. To quantify the effect of the mutant on the catalytic efficiency, we observed a previous work from our group about the study of the regulation mechanism of M3RA on its native state, where we defined a baseline for the energy barrier with a value of 25 kcal/mol [21]. This reference was used to differentiate between the catalytically efficient and the inactive enzymes, with barriers below and above that energy baseline, respectively. According to the experimental data, the free-energy barrier for the methyl-transfer process was close to 20 kcal/mol [19,29,30,31,32]; therefore, it was reasonable to define the system as catalytically inefficient or even inactive if it showed a decrease of four orders of magnitude of the velocity constant (k_cat_), which corresponded to an increase from 20 kcal/mol up to 25 kcal/mol.

**Figure 1:**
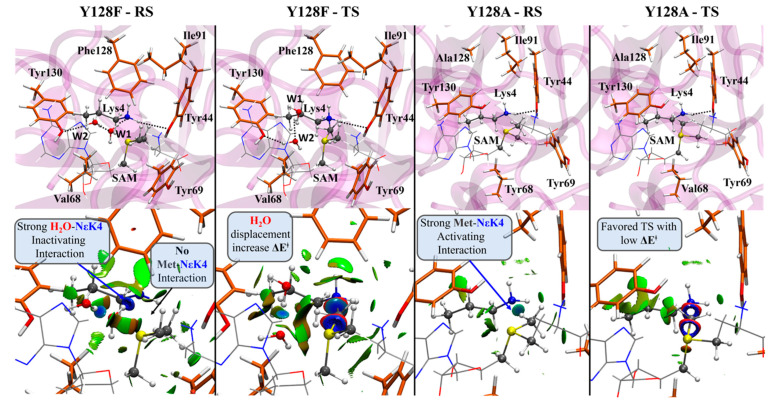
Structural representation of the catalytic site at RS and TS for the mono-methyl transfer reaction performed by the Y128F and Y128A mutants. Below, there is a graphic representation in real space of the non-covalent interactions occurring between the methyl group and the enzymatic environment, showing the mechanism for the inactivation of the Y128F mutant (ΔE^≠^ stands for barrier heights; enzyme’s carbon—orange; substrate and cofactor carbon—gray; red—oxygen; blue—nitrogen; white—hydrogen; and the isovalue used as cutoff for the surface was defined as 0.5).

The average values associated with the barrier heights, reaction energies, and a set of selected geometric parameters and interactions for Y44F and Y128F mutants are listed in Table 1 and Table 2. To observe the full data list for every system studied in the present paper, refer to the Supplementary Materials, Tables S2–S13. The data obtained from the calculations resulted in mean energy barriers (∆E^≠^) of 26.4 ± 9.9, and 37.7 ± 13.5 kcal/mol for Y44F and Y128F, respectively, both higher than the 18.9 ± 5.3 kcal/mol of ∆E^≠^ calculated in our previous work for the native enzyme [21]. In general, both mutants led to an inactive system, although Y128F was more impaired than Y44F because of the greater increase in ∆E^≠^, showing that its mutation affected, to a greater extent, the catalytic site. The average value of the C···N distance at the RS for the native system was 3.19 ± 0.23 Å [21], while for Y44F and Y128F mutants they were 3.77 ± 0.61 and 4.16 ± 0.68 Å, respectively, which correlated with the ∆E^≠^ values obtained for the two mutants. Regarding the TS, the mean values of the S–C distance showed a slightly longer bond than that observed for the native enzyme (2.30 ± 0.04 Å): 2.34 ± 0.10 and 2.37 ± 0.12 for Y44F and Y128F, respectively. This suggested that for the mutants, the enzyme needed to stretch the S–C bond to a greater extent to reach the TS region compared to the case of the native enzyme. This could be partially responsible for the higher ∆E^≠^ value. The Y44F mutation did not alter or impair the Y128-Nε(K4) interaction; the same was observed for Y128F, where the Y44-Nε(K4) interaction remained unaffected. However, both mutations modified the oxyanion-ring structure that stabilized the positive charge on the transferred methyl group, as shown by the data listed in Table 2. The remotion of the hydroxyl group from Y128 in the Y128F and Y44F mutants induced an important modification in the interaction pattern inside the catalytic site, where even the oxy-anionic ring did not completely interact with the methyl group and the substrate lysine, leading to what seemed to be an open conformation of the rest of the enzymatic machinery, as depicted in Figure 1. As a consequence of this open conformation, one of the most important phenomena observed for the impaired MLL3 mutant enzymes occurred: the entry of water molecules to the catalytic site that interacted with Nε(K4), standing between the reacting fragments, the methyl group, and the Nε(Lys4) atom. As detailed in our previous work [21], there was a highly stable and well-defined chain of water molecules in charge of deprotonating the lysine substrate before the methyl-transfer process. However, during the methyl-transfer process, there was only one water molecule forming part of the catalytic machinery in the native enzyme that interacted with Y128 and formed part of the oxy-anionic ring involved in the stabilization of the charge of the methyl group during its transfer. From an electronic point of view, the abnormal incorporation of water molecules into the binding site caused the sequestering of the electron pair from the Nε(K4), forming a strong hydrogen bond with one of the water molecules. To prove the existence of this interaction, we used the NCI method on the obtained structures. The results are shown in the bottom panels of Figure 1. NCI demonstrated the existence of the strong hydrogen bond between Nε(K4) and the water molecule, as denoted by the blue surface between the interacting parts. This hydrogen bonds impeded the methyl-group transfer to K4 lysine. The NCI surfaces also allowed us to identify that there was no interaction between the Nε(K4) atom and methyl group; the presence of water molecules distorted the reaction axis, changing the orientation of K4 with respect to the cofactor. In general, the rise in ∆E^≠^ was related to the need to desolvate the lysine amino group prior to the S_N_2 attack, which explains the impairment of the catalytic activity in these two mutant enzymes. A visual inspection of the water-molecule chain shows that they interacted with Y130, suggesting that this residue may work as a gating mechanism to avoid the access of water molecules after the deprotonation of the lysine substrate H^+^Nε(K4) necessary for the methyl-transfer process. Y130 is part of the oxy-anionic ring, and it is the impairment in the Y130–Met interaction that causes the access of the water molecules to the catalytic site. In fact, from Table 2, it is possible to appreciate an increased Y130–Met average distance from 3.11 ± 0.47 Å in the native enzyme to 4.64 ± 1.22 Å in the Y128F mutant. This revealed an important change in the Y130 conformation, in line with what has been suggested above, explaining the increased impairment observed for this mutant with respect to Y44F.

Another significant change observed for these mutants was the impairment of the Y69-OH···OOC-SAM interaction due to the incorporation of water molecules inside the binding site, which were located between these two moieties. In both mutants, Y44F and Y128F, the V68-Nε(K4) distance was shortened with respect to the native enzyme. A specific interaction that involved only the Y44F mutant occurred between the aromatic ring of F44 and the backbone of Y69, which seemed to destabilize the catalytic site conformation and was probably responsible for the Y69-OH···OOC-SAM elongation for this mutant. As previously established, Y44 was involved in the stabilization of the catalytic site and used as a regulator mechanism for the enzyme [21]. Apparently, the loss of its hydroxyl group caused the disorganization of part of the catalytic site, therefore decreasing the catalytic efficiency of the enzyme.

To determine the effect of the remotion of the phenyl ring from Y44 and Y128 in the mono-methyl transfer and later in the product selectivity process, we proceeded to mutate both residues to alanine, namely, Y44A and Y128A. The resulting ∆E^≠^ showed an interesting outcome, where Y44A remained as an inactive system but Y128A became a fully functional enzyme with a ∆E^≠^ of 20.3 ± 3.1 kcal/mol. From Table 2, it is possible to determine that the differences that led to the different ∆E^≠^ between Y44A and Y128A were, on the one hand, shorter between the Y128-Nε(K4) in Y44A when compared to the analogous interaction of Y44-Nε(K4) in Y128A, which in Y44A pulled the K4 substrate away from the methyl group. On the other hand, a significant difference was observed between the Y69-OH···OOC-SAM distance, which was shorter for Y128A than for Y44A. This reinforced the relevance of the presence of Y44 in the system to keep the structure of the SAM binding site, and thus the proper positioning of the methyl group to be transferred. These observations were supported by the analysis of the main geometric parameters associated with the reaction axis, where the C–N distance at both RS and TS of Y128A were almost the same as that obtained for the native system, with values of 3.19 ± 0.10 and 2.20 ± 0.05 Å, respectively. This denoted that despite the slight differences observed for Y128A and the native enzyme in the interactions of the oxy-anion ring with the methyl group, this mutant was almost equally catalytically efficient than the native enzyme. These results raise the question about why the remotion of the hydroxyl from Y128 leads to an inactive enzyme; however, the entire removal of the residue leaving an alanine produces an active enzyme, thus causing us to question the role of Y128 in the enzyme. According to the obtained structures, the hydroxyl group of Y128 helped to keep the tyrosine in a fixed position at the binding site; however, the lack of this group when having F128 led to a reorganization of the binding site by steric and electronic effects, which actually led to the conformational change of Y130 allowing the access of water molecules to the catalytic site. The removal of this residue avoided these conformational changes and reactivated the enzyme. Then, this again raised the question about the role of this residue in the catalytic site and, as revealed in the following sections in this study, it was related to the product selectivity of the enzyme. Specifically, when changing from Y128F to Y128A, we found that in the latter, Y130 adopted a position closer to the methyl group occluding the access of water molecules to the active site, as shown in Figure 1, exposing the conformational change previously mentioned. This leaves the electron pair in Nε(K4) free to be attacked by the methyl group, where the Met-Nε(K4) interaction is clearly identified by the blue surface, denoting a strong, attractive interaction according to the NCI index represented between the interacting parts.

In our previous work, we demonstrated that, in general, there was a linear correlation between the activation energy ∆E^≠^ and distance traveled by the methyl group that was transferred [21]. However, in the present case and as shown in Figures S2 and S3 and discussed in Section S2 in the Supplementary Materials, this linear correlation was not always present for these mutants. This denotes that there are additional factors involved in the control of the ∆E^≠^ as the entrance of water molecules to the binding site and the energy penalty needed to desolvate the reaction fragments, which clearly contributes to the increase in the energy barrier observed for some of the mutants.

### 2.2. Role of Y69F and Y130F Mutants in the Substrate-Inactivation Process

**Figure 2:**
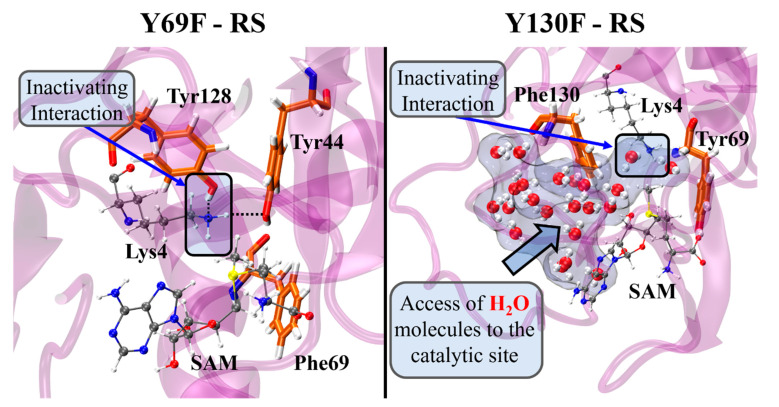
Structural representation of the inactivation mechanism of Y69F and Y130F mutants. (**Left**) The Y69F mutation leads to a conformational change of K4 that leads to the interaction of Y128 with the electron pair from NεK4 inactivating the substrate. (**Right**) The Y130 mutation opens a cavity between Y130 and SAM cofactor, which allows the access of water molecules to the catalytic site, where one molecule sequesters the electron pair from NεK4 avoiding Met attack.

The removal of the hydroxyl group from Y69 involves the loss of an anchor point for the SAM cofactor because, in the native enzyme, the carboxylic acid from SAM interacts with the hydroxyl group of this residue. In this context, the major effects of the mutation of Y69F are expected to be focused on the lack of stability in SAM binding and, therefore, the proper positioning of the methyl group. In fact, the Y69F mutant showed an increase of ∆E^≠^ to 32.7 ± 16.6 kcal/mol, an energetic barrier significantly larger than that of the native enzyme (18.9 ± 5.30 kcal/mol), pointing out that this mutated system was not catalytically viable. The increase in the barrier can be justified by the increase in the mean value of the C···N distance, from 3.19 ± 0.23 Å for the native enzyme to 4.01 ± 1.11 Å for this mutant. To establish the changes in the catalytic site that may lead to the lengthening of this distance, we proceeded by comparing the differences between the native and mutant enzymes in terms of the main geometric parameters, including the interactions between substrate K4, the methyl group, and the catalytic site, with the data listed in Table 1 and Table 2. According to this, there was a clear lengthening of the Y44-Nε(K4) interaction distance in both the RS and TS, whereas for the Y128-Nε(K4) interaction, the lengthening was observed only at the TS. The lack of stable positioning of the SAM cofactor in its binding site positioned the methyl group away from its normal location, pulling the K4 substrate away from the tyrosines, thus lengthening the distance of K4 with Y44 and Y128. While the conformations that led to lower ∆E^≠^ values for the methyl transfer showed that both Y44 and Y128 stabilized the substrate by acting as proton acceptors of Nε(K4), in those configurations showing an increase of ∆E^≠^, Y128 acted as a proton donor to Nε(K4), as shown in Figure 2 (left panel). This involved the inactivation of the Nε(K4) electron pair as it was sequestered by Y128, explaining the observed increase of ∆E^≠^. In general, the distances between the residues from the oxy-anion ring and the methyl group were longer than in the native enzyme (the distances are shown in Table 2), whereas the V68-K4 interaction was slightly shortened, probably to compensate for this effect. The longer interactions with the methyl group may have been responsible for the increase of ∆E^≠^ and were caused by the change in the structure of the catalytic site due to the lack of stability of SAM at its binding site. Additionally, there seemed to be an increase in the number of water molecules at the catalytic site as is discussed in the proceeding sections of the paper, although this was not as prevalent as that observed for Y44F and Y128F.

The Y130F mutant led to a ∆E^≠^ increase concerning the native enzyme, with a value of 34.8 ± 11.2 kcal/mol, as listed in Table 1, which denoted that this mutation inactivated the enzyme. The main geometric parameter identified as responsible for the inactivation of the enzyme was the increase in the mean C···N distance from 3.19 ± 0.23 Å for the native enzyme to 3.74 ± 0.78 Å for the Y130F mutant. This change was mainly attributed to the increase in the distances of Y44-Nε(K4) and Y69-SAM interactions that entailed the conformational destabilization of both the K4 substrate and methyl group. In addition, an increase in the I91-Met distance was observed for Y69F. Meanwhile, there was a shortening of the V68-Met interaction distance that probably compensated for the lack of a Y130-Met anchor point in the oxy-anion ring.

One of the most important findings regarding this mutant was that the removal of the hydroxyl group from Y130 induced a conformation change in this residue, which opened the access of the water molecules from the outside of the enzyme, as shown in Figure 2, and thus inactivated the K4 substrate as previously observed. This is in line with the inactivation mechanism described for Y44F and Y128F, where Y130 seems to play a key role in the occlusion of water molecules from the catalytic site. For Y128F, the conformational change of Y130 is related to a displacement of the backbone of F128 that pulls Y130 into an open conformation, leading to a greater increase in the ∆E^≠^ (37.7 ± 13.5 kcal/mol) when compared to Y44F, which was marginally inactive with a mean ∆E^≠^ value of 26.4 ± 9.9 kcal/mol. These results corroborate the role of Y130 in the exclusion of water molecules, closing the space between the SAM cofactor and the lysine substrate to the solvent and acting as a wall for the channel through which the methyl group is transferred.

### 2.3. Di- and Trimethyl-Transfer Processes: The Effect of Mutations on Product Selectivity

**Figure 3:**
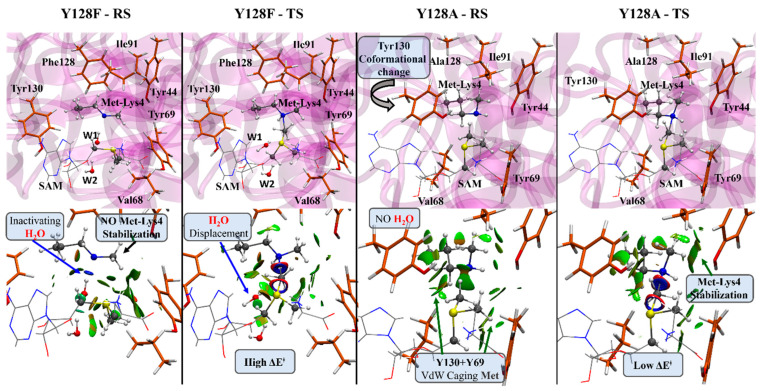
Structural representation of the catalytic site at RS and TS for the di-methyl-transfer reaction performed by the Y128F and Y128A mutants. Below, there is a graphic representation in real space of the non-covalent interactions occurring between the methyl group and the enzymatic environment, showing the mechanism for the reactivation of the enzyme through the Y128A mutant (ΔE^≠^ stands for barrier heights; enzyme’s carbon—orange; substrate and cofactor carbon—gray; red—oxygen; blue—nitrogen; white—hydrogen; and the isovalue used as cutoff for the surface is defined as 0.5).

**Figure 4:**
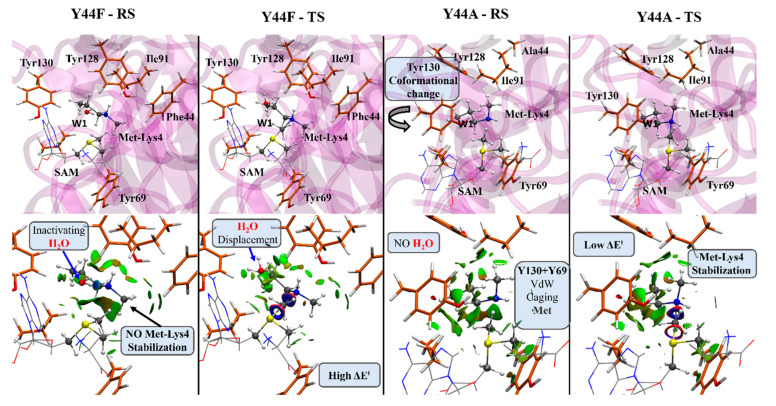
Structural representation of the catalytic site at RS and TS for the di-methyl-transfer reaction performed by Y44F and Y44A mutants. Below, there is a graphic representation in real space of the non-covalent interactions occurring between the methyl group and the enzymatic environment, showing the mechanism for the reactivation of the enzyme through the Y128A mutant (ΔE^≠^ stands for barrier heights; enzyme’s carbon—orange; substrate and cofactor carbon—gray; red—oxygen; blue—nitrogen; white—hydrogen; and the isovalue used as cutoff for the surface is defined as 0.5).

The experimental background previously described in this study points out the relevance between the number of tyrosines at the catalytic site and the product specificity. According to the previous discussion, there were cases when mutating one of the tyrosines at the binding site of mono-methyltransferase enzymes into smaller residues, such as valine, cysteine, or even phenylalanine, derived a gain of function with new capacities to di- or even tri-methylate the lysine. However, there was also the risk of inactivating the enzyme, as the role of each tyrosine in the catalytic site was still a subject of debate, and some tyrosines seemed to be essential to maintain the basal catalytic activity. To explore the role of each tyrosine further and to provide insights into the mechanisms by which product selectivity can change in MLL3 through specific mutations, we performed the mutations of Y44 and Y128 into phenylalanine and alanine. The results are presented in the form of a selected set geometric parameters, interaction distances, and energies listed in Table 3 and Table 4. The mutation into phenylalanine generated, in both cases of Y44F and Y128F, inactive systems towards the dimethyl-transfer process, with averaged ∆E^≠^ values of 26.4 ± 7.2 and 44.1 ± 16.7 kcal/mol, respectively. In both cases, we observed that a water molecule interacted with Nε(K4), inactivating the electron pair as previously observed for the mutants that were catalytically inactive for mono-methylation. The greater increase in the barrier height observed for Y128F is related to a major increase in the mean C···N distance from 3.19 ± 0.23 in the native to 5.55 ± 0.75 Å in the mutant. In general, there was a lengthening of the interaction distances between the methyl group and oxy-anionic ring at the TS of Y128F, as seen in Table 3 and Table 4. Additionally, an increase in the Y69-SAM distance with respect to the mono-methyl-transfer reaction was observed, losing the ability to keep the methyl group at a fixed position close to the substrate, and thus contributing to the significant increase in the C···N distance and ∆E^≠^. For the case of Y128F, the changes in the distances between the methyl group and oxy-anionic ring were mostly due to the incorporation several water molecules into the catalytic site, two of which are shown in Figure 3. This contributed to the increase in the distances between the interacting parts and the enzymatic machinery and, also, to the inactivation of the substrate. Interestingly, the conformation of Y130 inside the binding site changed completely with respect to the native enzyme, where the latter was located close to the methyl group and the Y128F mutant was located at ~8.0 Å from the methyl group, thereby opening the access of the water molecules located at the vicinity of the binding site. For Y44F (results shown in Figure 4), the inactivation mechanism was the same, with a water molecule interacting with the Nε(K4). In both mutants, the methyl group of the MetK4 substrate pointed towards the aromatic ring of residue 44 (Y or F). The main conformational difference between Y44F and Y128F was the hydrogen bond formed between Y128 and the proton from Nε(K4) in the former. The proton in the Y128F mutant did not interact with any residue or water molecule.

The Y44A and Y128A mutants, on the other hand, presented a remarkable result. The replacement of the original residues by alanine resulted in two fully active systems towards dimethyl-transfer with averaged ∆E^≠^ values of 15.5 ± 6.0 and 15.8 ± 3.1 kcal/mol for Y44A and Y128A, respectively. This result is noticeable as MLL3 is only capable of mono-methylate in its native state. The geometric parameters of the reaction axis, namely, the C···N and S···C distances together with the S···C···N angle, were all very close to the values obtained for the native enzyme for the mono-methyl transfer; values listed in Table 1. The C···N distances were even slightly shorter for the two mutants with average values of 3.16 ± 0.38 Å for Y44A and 3.14 ± 0.17 Å for Y128A. An important structural feature in the two mutants was the conformation change of Y130 with respect to the mutants with phenylalanine, where the proper positioning of Y130 close to the methyl group to be transferred, shown in Figure 3 and Figure 4 for Y128A and Y44A, respectively, seemed to be essential to maintain proper catalytic activity, preventing the entrance of water molecules into the active site. In Y128A, the methyl group from the MetK4 substrate was accommodated in a cavity formed by the side chains of I91, Y130, and F71, all forming a hydrophobic pocket. In the case of Y44A, a similar cavity was formed, but with the aromatic ring from Y128 taking part of this binding site, as shown in Figure 4. From the reaction energies listed in Table 3, it is possible to denote an important increase in the exothermicity when transitioning from Y44F and Y128F to Y44A and Y128A, which evidences the higher degree of stabilization of the products of the reaction for these two new mutants. Therefore, the main properties that make Y44A and Y128A active compared to Y44F and Y128F towards dimethyl-transfer are the complete absence of water molecules in the catalytic site mediated by Y130 and the formation of a hydrophobic cavity capable of accommodating the methyl group from the MetK4 substrate.

**Figure 5:**
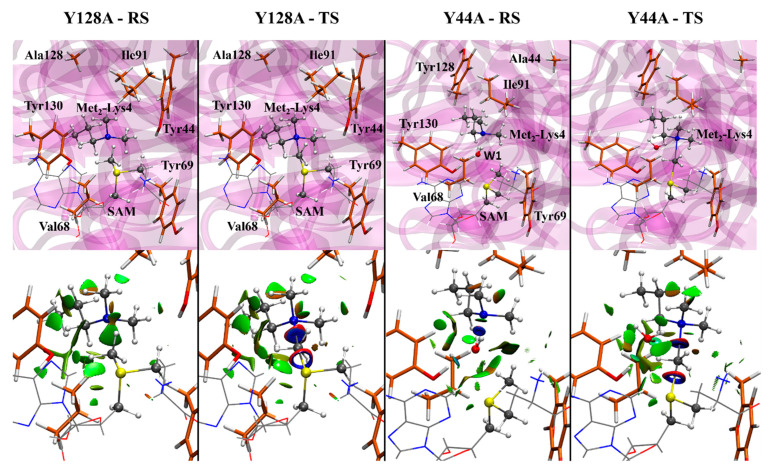
Structural representation of the catalytic site at the RS and TS for the tri-methyl-transfer reaction performed by the Y128A and Y44A mutants. Below, there is a graphic representation in real space of the non-covalent interactions occurring between the methyl group and the enzymatic environment, showing the stabilization at the RS of the dimethyl lysine at the binding site for Y128A aided by the mutation of the tyrosine by alanine (enzyme’s carbon—orange; substrate and cofactor carbon—gray; red—oxygen; blue—nitrogen; white—hydrogen; and the isovalue used as cutoff for the surface is defined as 0.5).

Regarding the product specificity of KMTs, the tri-methylated lysine is considered one of the most active epigenetic marks in terms of gene activation or silencing. In this sense, testing the possibility that the mutants of MLL3 are capable of tri-methylate K4(H3) becomes crucial for the understanding of pathologies that may involve such mutations. As we observed, only those mutants where tyrosines were mutated by alanine showed such a gain of function for di-methylation activity. Therefore, we tested the catalytic efficiency of Y44A and Y128A mutants towards trimethyl-transfer. The results show that while Y44A is inactive, with a ∆E^≠^ of 34.6 ± 11.7 kcal/mol, the Y128A mutant shows an average ∆E^≠^ of 15.2 ± 5.9 kcal/mol (see Table 3), which indicates that it is not only active, but it even shows an energy barrier lower than that found for the mono-methyl-transfer reaction performed by the native enzyme. From a thermodynamic point of view, the more negative reaction energy ΔE° observed for Y128A evidenced the higher stabilization rate of the product formed by Y128A compared to Y44A. From our results, it can be suggested that the mutation of Y128 to alanine originates an enzyme capable of generating mono-, di-, and tri-methylated K4, while Y44A is only able to use mono-methylated K4 to add a second methyl group, without mono- and tri-methylation activity. The main structural parameter that distinguishes between Y44A and Y128A for the tri-methylation was the distance between NεK4 and the corresponding non-mutated tyrosine. Where, for Y128A, the interaction distances to Y44 were 2.61 ± 0.17 Å (RS) and 2.60 ± 0.17 Å (TS), for Y44A, the interaction distances to Y128 were of 3.94 ± 0.75 Å (RS) and 4.03 ± 0.87 Å (TS), showing the lack of interaction between Y128 and NεK4 in Y44A. These results also mark the dependence on Y44 to keep the methylation activity in most cases. As expected, inside the catalytic site of Y44A, there was a water molecule interacting with Nε(K4), inactivating the enzyme. Considering that the substrate for the tri-methylation is a di-methyl lysine, we observed that for the Y128A mutant, there seemed to be a greater stabilization of the di-methyl moiety of this substrate as denoted by the larger extent of the NCI index observed for this mutant and represented by the green surface surrounding the di-methyl moiety in Figure 5. Here, one methyl group points towards the Y44 hydroxyl group and the other points to the methyl group of A128. Meanwhile, Y44A showed that this di-methyl moiety was less stable inside the binding site, adopting different positions in the binding site along the 25 conformations studied. This was probably due to the presence of water molecules in the active site of Y44A. From a thermodynamic point of view, the more negative vales of ΔE° observed for Y128A evidence the higher stabilization of the tri-methylated product formed by Y128A compared to Y44A.

### 2.4. Inactive Versus Active MLL3 Mutants

At this point, the main mechanism for the activation and inactivation of the enzyme depends on the accessibility of water molecules to the catalytic site. To evidence the predominance of this mechanism in the systems studied through QMMM calculations, we calculated the percentage of systems from the 25 RS and TS conformers selected for the calculations for each mutant, in which there was at least one water molecule interacting with Nε(K4) from the substrate lysine. The results are presented in Table 5. For the mono-methyl transfer, 84% of the RS conformers of Y128F presented a water molecule interacting with Nε(K4), while in the case of the Y128A mutant, all reactant conformers were free of water molecules sequestering the electron pair from the lysine. The decrease in the percentage of conformers presenting water molecules observed for the Y128F mutant when moving from RS to TS was the result of the fact that the water molecule was displaced by the methyl group during the attack. A similar trend was observed for the di-methylation process, where the activation observed when moving from Y128F to Y128A required a decrease from 60% of water molecules interacting with Nε(K4) to only 8% in order to be active. A low percentage was also observed for the Y128A mutant active towards tri-methylation with no conformers being inactivated. The removal of the aromatic ring when mutation by alanine involved the loss of the steric effect allowed the Y130 residue to adopt its optimal conformation close to the methyl group from SAM, as explained above, which is required to avoid the presence of water molecules in the active site. Our results reveal that the removal of the phenyl ring from Y128 leads to a change in the product specificity towards di- and tri-methylation in addition to mono-methylation activity. For the case of Y44F, we also observed a conformational change in the side chain of Y130 to an open conformation, permitting the access of water molecules to the catalytic site. The Y44A mutant induced an effect similar to that observed for Y44F. These results also mark the relevance of the presence of Y44 to keep the methylation activity.

**Figure 6:**
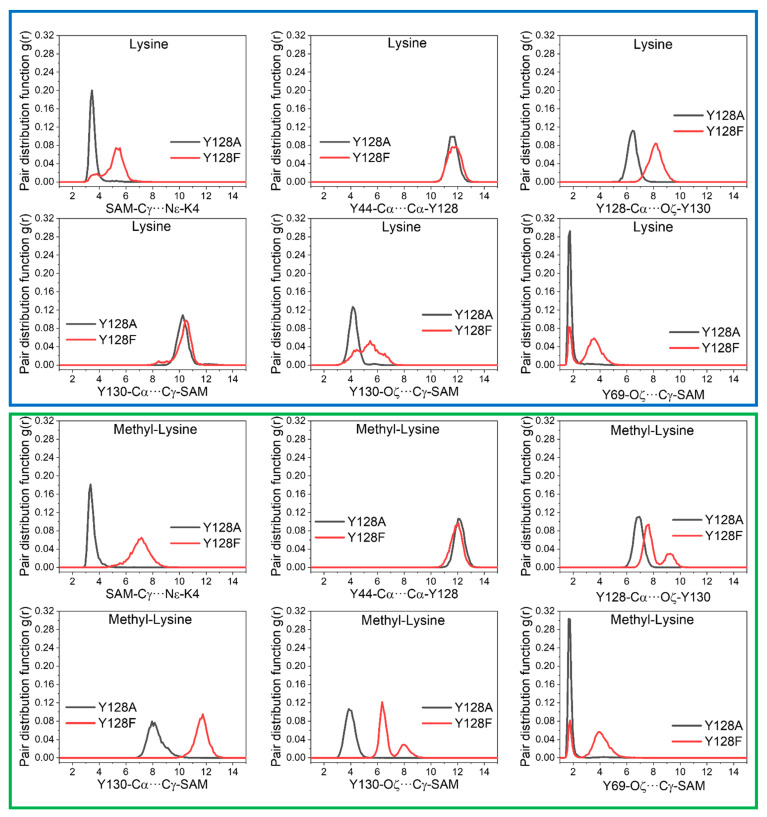
Plots of the pair-distribution functions for selected distances associated with the reactivation mechanism sampled along 50 ns of molecular dynamic simulations of Y128F and Y128A mutants with the unmethylated and mono-methylated lysines. In the blue box are the interactions involving Y128F and Y128A for the systems sampled to perform the first methylation process; in the green box are the interactions involving Y128F and Y128A for the systems sampled to perform the di-methylation process.

To support the evidence revealed at this point about the inactivation and gain-of-function molecular mechanism for the MLL3 mutants studied in this paper, we proceeded by generalizing the main conclusions obtained through the QMMM calculations analyzing the radial-pair-distribution function of several conformational parameters obtained from the MD simulations. These were used to understand the correlation between chemical modifications due to the mutation and its impact on the conformation of certain residues at the catalytic site, in particular, Y130. To provide a means to understand the reactivation of the enzyme towards mono-methylation, we compared Y128F with Y128A, which represented a decrease in the ∆E^≠^ from 37.7 to 20.3 kcal/mol; data shown in Figure 6. As expected from our previous analysis, there was a clear increase in the C···N distances in the inactive enzyme. Despite the mutation towards phenylalanine or alanine, there were no major changes in the location of Y44 with respect to F128 or A128 as denoted by the similar distances between their Cα during the simulation. Nevertheless, Y130 showed a clear conformational change, evidenced by the distance between its hydroxyl oxygen (Oζ) atom to the Cα of Y128 residue that, at this point, seemed to have the largest influence on Y130 conformation due to its proximity in sequence. This conformational change was also evidenced by the increase in the distance between the Oζ from Y130 and the carbon (Cγ) from the methyl group, from which it should actually remain close to the cavity between this residue and SAM, thereby avoiding the access of water molecules. Similarly, to shed light onto the gain-of-function mechanism towards di-methylation, we compared Y128F and Y128A, which represented inactive and active states, respectively, for the addition of a second methyl group to K4. The longer C···N distances in the inactive enzyme were also observed for this case. Regarding the main conformational changes involved in the activation of the enzyme towards di-methylation, Y130 showed a similar behavior as in the reactivation of the enzyme to mono-methylate K4. This implied that the positioning of Y130 with respect to the methyl group confirmed that Y130 needed to adopt a conformation located close to the methyl group in the active systems.

**Figure 7:**
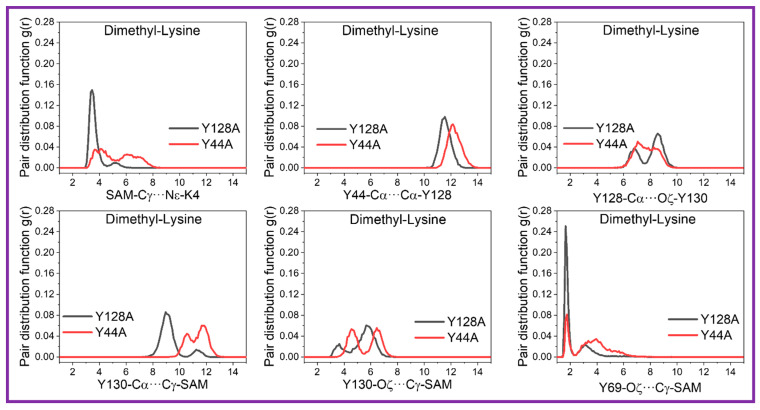
Plots of the pair-distribution functions for selected distances associated with the reactivation mechanism sampled along 50 ns of molecular dynamic simulations of Y128A and Y44A mutants with the di-methylated lysine. In the box are the interactions involving Y44A and Y128A for the systems sampled to perform the tri-methylation process.

It is remarkable how the conformation of Y130 determined the catalytic efficiency, which was achieved by excluding water molecules from the catalytic site, and also how its behavior depended on Y44 and Y128. To explore the role of Y130 in the tri-methylation process, we proceeded with an analogous analysis of the interaction distances, using Y44A as the model for an inactive system and Y128A for the model of an active state of the enzyme towards tri-methylation. According to the results presented in Figure 7, by triangulating the position of Y130 using the Cα from Y128 as a reference point, there seems to be a higher distribution for the inactive system, whereas the active state shows two distinctive positions of the hydroxyl group from Y130. However, the main indicator of the conformational change of Y130 is associated with the observation that the Oζ from Y130 is close to the methyl group in the active system, revealing that in general the position of Y130 plays a crucial role in the catalytic efficiency of the activity of mono-, di-, and tri-methylate K4. The integration of the main results found in this work converged in a common mechanism for all the active mutants, where the gain-of-function mutant enzymes always depend on the occlusion of water molecules performed by the adoption of a specific conformation of Y130 close to the methyl group from SAM. At the same time, the inactivation mechanism involved the conformational change of Y130 that permitted the access of water molecules to the catalytic site with the consequent sequestering of the electron pair from Nε(K4) in the substrate lysine.

**Figure 8:**
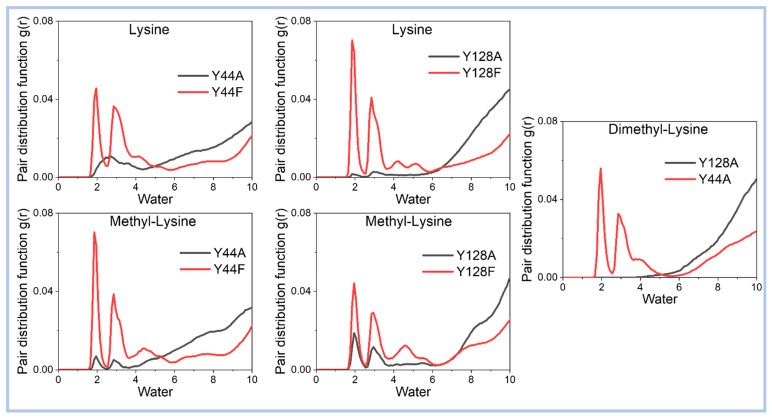
Plots of the pair-distribution functions for the interaction between the nitrogen from the lysine substrate (Nε(K4)) and any water within a radius of 10 Å sampled along 50 ns of molecular dynamic simulations for the unmethylated, mono-methylated, and di-methylated lysine, for all the mutant states here studied.

Finally, to support the relation between the conformational requirement of Y130 in the active systems and the exclusion of water molecules from the active site, we determined the radial-pair-distribution function between Nε(K4) and any water molecules within a range of 10 Å along the 50 ns of MD simulation. The results presented in Figure 8 show the analysis for the unmethylated, mono-methylated, and tri-methylated lysine. For the unmethylated lysine, in both Y44F and Y128F mutants, we observed the presence of water molecules at the interaction distance from Nε(K4), which was higher for Y128F in line with the greater increase of ∆E^≠^ for this mutant. For the Y128A mutant, there was clear, water exclusion with almost no water near Nε(K4), whereas Y44A still showed the entrance of water molecules, which explained why this system was still inactive after mutating Y44 by alanine. For the system with the mono-methylated lysine, there was also a decrease in the access of water to the binding site when mutating by alanine instead of phenylalanine, although there was still some incorporation of water molecules to the site. For di-methylated lysine, the change was more drastic, where no water was able to access to the binding site for the Y128A mutant, which was the active enzyme, while the Y44A system showed a high proportion of water molecules at interacting distance from Nε(K4), in agreement with its inactive state.

## 3. Materials and Methods

### 3.1. System Setup and Conformational Sampling

The coordinates for the trimeric functional system MLL3-ASH2L-RBBP5 (M3RA) enzyme in a complex with an H3 segment as a peptide substrate was obtained from the crystal structure found in the Protein Data Bank with PDB code 5F6K [19]. To define the protonation states of the titratable residues, the PropKa 3.4.0 program was used [33,34] at a pH of 7.8, in line with the activity assays presented by Li et al. [19]. To model the mutant states of the enzyme, the MUTATE plugin from VMD (v1.9.1) was used [35]. In this context, six mutants were modeled: Y44F, Y69F, Y128F, Y130F, Y44A, and Y128A. These mutants were selected as their mutation may provide the necessary space to accommodate the methyl groups for successive methyl-transfer reactions. In fact, di-methylation activity was evaluated for mutants Y44F, Y128F, Y44A, and Y128A, while tri-methylation activity was evaluated for Y44A and Y128A. Each enzymatic model was embedded in a rectangular box of TIP3P water molecules, followed by the addition of enough ions (Na^+^ and Cl^−^) to reach a concentration of 0.05 mol/L and to ensure the charge neutrality of the system. All the simulations were performed using the NAMD program version 2.9 [36], considering periodic boundary conditions. The parameters for the system were obtained from the CHARMM22 force field for the protein [37,38], whereas, for the SAM cofactor, these were obtained from a previously published set of parameters [39]. The molecular dynamics simulations were run using a time step of 2 fs. The Langevin piston Nosé–Hoover method kept the temperature and pressure at 300 K and 1 atm, respectively [40]. The bonds involving hydrogen atoms were restrained by the SHAKE algorithm implemented in the NAMD code [41]. As an equilibration protocol, we first allowed the water molecules to gather around the protein surface and fill the cavities, while the protein was kept fixed by initially minimizing the 2000 steps of conjugate gradients, followed by 0.1 ns of MD simulation. Then, three stages of successive MD equilibration calculations of 0.1 ns were performed with decreasing restraints using force constants of 5.0, 2.0, and 0.1 kcal/mol·Å^2^, respectively, applied to the protein, substrate, and cofactor. Then, 50 ns of production dynamics were run to generate the conformational sampling for each state of the enzyme that would be used as the starting structures for the QM/MM calculations. According to this, in the present work, twelve 50 ns MD simulations were run, six to test the catalytic efficiency towards the mono-methyl transfer after mutation, and six more to analyze the roles of Y44 and Y128 in the product specificity of MLL3 and how its mutations may have led to the impaired or gain-of-function states of the enzyme.

### 3.2. Setup of QM/MM Calculations

The main goal was to keep as much of the protein as possible included in the model system to capture the chemical role of the enzymatic environment in the catalytic reaction. To achieve that goal, the QM/MM approach provided the best balance between the system size and efficiency, offering a realistic representation of the system. To obtain the initial structures for the QM/MM calculations, 25 snapshots were selected from the 50 ns of production dynamics for each state of the enzyme. Each snapshot was selected within a window of 2 ns, picking the shortest SAM-C···Nε(K4) distance and the largest S···C···N angle within each sampling window, ensuring a separation of at least 500 ps between two conformations obtained from adjacent sampling windows. Such a selection method was preferred to the random selection as it captured the structural features that characterized the active system that would actually have access to catalyze the reaction. After the conformer selection process, the QM/MM model was setup by considering the full enzyme and a layer of 5 Å of water molecules surrounding the protein. The active region for the optimization procedure was defined as all residues and water molecules within 8 Å from the sulfur of the SAM cofactor. The QM region was composed of the SAM cofactor, the substrate lysine H3K4, the tyrosine tetrad in its mutant form, and in some cases a water molecule that was incorporated whenever a water molecule was within 3 Å from H3K4. All the residues included in the QM region were truncated between Cα and Cβ, while for SAM, the sulfur and methyl groups directly bonded to it were considered as part of the QM region.

All geometry optimizations were conducted using B3LYP via the density-functional theory [42,43], which has proven to provide accurate results for other methyltransferases [44,45,46]. For the optimizations, the all-electron def2-SVP basis set was used [47], which was followed by energy correction by using the def2-TZVPP basis set [48]. All DFT calculations included the DFT-D3 method with the Becke–Johnson damping function [49,50,51].

The transition-state search for the methyl-transfer process was performed by scanning along a reaction coordinate, namely, the difference in distances between [Nε(Lys4)···Cγ(SAM)—Sδ(SAM)···Cγ(SAM)], labels depicted in Scheme 1, as suggested by Bruice et al. [52]. Once a maximum was observed on the potential energy surface, transition-state (TS) optimization was performed using the dimer method [53]. All QM/MM calculations were performed using the ChemShell suite [54], coupled with Turbomole v7.3 [55] to perform the electronic-structure calculations involving the QM region. On the other hand, the calculations of the MM region were performed with the DL POLY program as implemented in the ChemShell suite.

To obtain a qualitative picture of the contribution and topology of the non-covalent interactions occurring inside the catalytic site, we calculated the non-covalent interaction index (NCI) [56,57]. This provides a real-space interpretation of the non-covalent interactions between the reacting parts, namely, SAM and Lys4, with the enzymatic machinery. That allows for the identification of the stabilizing contributions rising from the interactions provided by the enzymatic structure to the reaction process. Additionally, it allowed us to differentiate between attractive and repulsive interactions. For interpretative purposes, the regions of the surface colored in blue denote strong stabilizing interactions, green indicates weak interactions usually associated with van der Waals interactions, and those colored in red are indicative of repulsive interactions. For brevity, we defined the reactant, transition, and product states as RS, TS, and PS, respectively.

## 4. Conclusions

The methylation of the N-terminal domain from histones represents a dynamic mechanism of epigenetic regulation due to its versatility in terms of the activation and inactivation of gene regulation together with the degree of activation depending on the product multiplicity. Herein, we explored through QM/MM calculations and molecular dynamics simulations the potential capacity of MLL3 to change its mono-methylation activity by modifying two key residues in charge of substrate anchoring, namely, Y44 and Y128. While our models showed that the mutation into phenylalanine of both residues led to inactive states of the enzyme, the mutation of Y128 to alanine (Y128A) resulted in an active enzyme for the mono-methylation of the substrate, whereas Y44A was inactive. However, the most remarkable result was that, according to our calculation, the Y128A mutant was actually able to di- and tri-methylate the substrate, which could cause diseases due to the increased range of products for this enzyme. Meanwhile, Y44A was only capable of di-methylating. According to this, we were able to suggest a proper mechanism for the inactivation and gain of function for this enzyme, in which Y130 showed a crucial role in the process. In this sense, we were able to expose how, when Y128 was mutated into phenylalanine, there was a conformational change of Y130 through steric effects, which opened the active site to water molecules and inactivated the lysine substrate by sequestering the electron pair of its nitrogen atom. However, the mutation into alanine eliminated that steric effect, allowing Y130 to restrict the access of water molecules and thus reactivate the enzyme. In addition, Y128A mutation provided access to the formation of a hydrophobic pocket capable of accommodating the methyl groups present in lysine during the di- and tri-methylation processes. Finally, it was possible to stablish the relevance of Y128 in the product specificity of the enzyme.

## Figures and Tables

**Table 1:** Barrier heights (ΔE^≠^), reaction energies (ΔE_1_°), and selected average geometric parameters with their corresponding standard deviations for the RS and TS conformations of the mono-methyl-transfer reaction in the native enzyme M3RA and mutants Y44F, Y128F, Y44A, Y128A, Y69F, and Y130F. Average activation and reaction energies are in kcal/mol, distances in Å, and angles in degrees (°).

System	M3RA ^a^	Y44F	Y128F	Y44A	Y128A	Y69F	Y130F
ΔE^≠^	18.9 ± 5.30	26.4 ± 9.9	37.7 ± 13.5	29.3 ± 9.6	20.3 ± 3.1	32.7 ± 16.6	34.8 ± 11.2
ΔE°	−10.7 ± 7.6	−14.2 ± 8.7	−5.5 ± 7.2	−5.4 ± 10.1	−7.5 ± 5.1	−5.4 ± 9.5	−5.3 ± 9.5
C-N(RS)	3.19 ± 0.23	3.77 ± 0.61	4.16 ± 0.68	3.43 ± 0.37	3.19 ± 0.10	4.01 ± 1.11	3.74 ± 0.78
C-N(TS)	2.20 ± 0.05	2.28 ± 0.06	2.37 ± 0.14	2.21 ± 0.07	2.20 ± 0.05	2.25 ± 0.10	2.23 ± 0.07
S-C(RS)	1.82 ± 0.01	1.82 ± 0	1.81 ± 0.01	1.82 ± 0.01	1.81 ± 0.01	1.82 ± 0.01	1.81 ± 0.01
S-C(TS)	2.30 ± 0.04	2.34 ± 0.10	2.37 ± 0.12	2.41 ± 0.15	2.3 ± 0.11	2.44 ± 0.15	2.36 ± 0.13
S-C-N(RS)	151.0 ± 15.7	136 ± 19.4	123.8 ± 17.0	126.2 ± 26.4	132.8 ± 16.7	144.2 ± 19.0	116.2 ± 34.3
S-C-N(TS)	170.9 ± 4.5	166.7 ± 5.3	158.2 ± 12.6	161 ± 18.8	169.7 ± 3.5	167.9 ± 6.2	163.8 ± 8.0

^a^ Data obtained from reference [21].

**Table 2:** Mean values for some selected interaction distances between the cofactor and substrate at the catalytic site, for M3RA, and the mutants Y44F, Y128F, Y44A, Y128A, Y69F, and Y130F for their respective RS and TS for the mono-methyl-transfer reaction. Distances are in Å and are provided with their corresponding standard deviations.

Systems	State	Y44-K4 ^a^	Y128-K4 ^a^	V68-Met ^b^	R89-Met ^b^	I91-Met ^b^	Y130-Met ^c^	Y69-SAM ^d^
M3RA ^e^	RS	2.30 ± 0.34	2.55 ± 0.39	3.25 ± 1.10	3.20 ± 0.41	3.19 ± 0.45	3.11 ± 0.47	1.57 ± 0.03
	TS	2.43 ± 0.49	2.48 ± 0.41	3.00 ± 1.06	3.22 ± 0.43	3.26 ± 0.34	2.93 ± 0.47	1.57 ± 0.03
Y44F	RS	---	2.31 ±0.42	2.66 ± 0.26	3.08 ± 0.63	3.77 ± 0.68	3.49 ± 0.75	4.73 ± 2.09
	TS	---	2.62 ± 0.71	2.65 ± 0.25	3.66 ± 0.58	3.43 ± 0.45	2.86 ± 0.50	4.65 ± 2.05
Y128F	RS	2.36 ± 0.28	---	2.60 ± 0.21	2.88 ± 0.49	3.25 ± 0.61	4.64 ± 1.22	2.78 ± 1.00
	TS	2.44 ± 0.39	---	2.59 ± 0.25	3.42 ± 0.51	3.04 ± 0.34	3.80 ± 1.05	2.68 ± 0.94
Y44A	RS	---	2.07 ± 0.36	2.53 ± 0.27	2.83 ± 0.38	3.31 ± 0.52	3.61 ± 0.71	1.99 ± 0.66
	TS	---	2.26 ± 0.26	2.46 ± 0.16	3.37 ± 0.81	3.29 ± 0.38	2.77 ± 0.50	1.97 ± 0.67
Y128A	RS	2.57 ± 0.64	---	2.87 ± 0.31	2.75 ± 0.35	3.14 ± 0.41	3.66 ± 0.52	1.70 ± 0.45
	TS	2.44 ± 0.59	---	2.54 ± 0.23	3.29 ± 0.40	3.05 ± 0.36	2.86 ± 0.39	1.69 ± 0.45
Y69F	RS	3.16 ± 1.52	2.54 ± 1.19	3.39 ± 1.02	3.70 ± 1.15	3.93 ± 1.32	3.13 ± 0.57	---
	TS	3.71 ± 1.83	3.21 ± 1.26	2.96 ± 0.68	3.68 ± 0.97	3.68 ± 0.76	2.54 ± 0.26	---
Y130F	RS	3.32 ± 1.04	2.78 ± 0.94	3.00 ± 0.57	3.15 ± 0.80	3.84 ± 1.35	---	2.28 ± 0.95
	TS	3.40 ± 1.38	2.85 ± 0.94	2.61 ± 0.21	3.72 ± 0.82	3.55 ± 0.49	---	2.13 ± 0.79

^a^ Distance between hydroxyl oxygen from Tyr and the closest proton from Lys4. ^b^ Distance between carboxyl oxygen obtained from the backbone of the residue and the closest hydrogen obtained from Met. ^c^ Distance between the hydroxyl oxygen obtained from Tyr and the closest hydrogen obtained from Met. ^d^ Distance between the hydroxyl group obtained from Tyr69 and the closest oxygen obtained from the carboxylic acid of SAM. ^e^ Data obtained from reference [21]. --- means no data in the column in the table.

**Table 3:** Selected average geometric parameters with their corresponding standard deviations for the RS and TS conformations of the di- and tri-methylation reactions in the mutants Y44F, Y128F, Y44A, and Y128A. Average activation (ΔE^≠^) and reaction energies (ΔE°) are in kcal/mol, distances in Å, and angles in degrees (°).

Systems	Y44F-diMet	Y44A-diMet	Y128F-diMet	Y128A-diMet	Y44A-triMet	Y128A-triMet
ΔE^≠^	26.4 ± 7.2	15.5 ± 6.0	44.1 ± 16.7	15.8 ± 3.1	34.6 ± 11.7	15.2 ± 5.9
ΔE°	−9.6 ± 5.4	−14.9 ± 5.1	0.6 ± 8.9	−12.9 ± 4.8	−4.6 ± 7.0	−17.3 ± 8.0
C-N(RS)	3.70 ± 0.60	3.16 ± 0.38	5.55 ± 0.75	3.14 ± 0.17	4.64 ± 0.83	3.25 ± 0.47
C-N(TS)	2.28 ± 0.05	2.22 ± 0.06	2.30 ± 0.08	2.21 ± 0.05	2.29 ± 0.09	2.26 ± 0.04
S-C(RS)	1.81 ± 0.01	1.82 ± 0.01	1.81 ± 0.01	1.82 ± 0.01	1.81 ± 0.01	1.81 ± 0.01
S-C(TS)	2.33 ± 0.08	2.28 ± 0.06	2.31 ± 0.09	2.29 ± 0.04	2.35 ± 0.10	2.28 ± 0.07
S-C-N(RS)	133.9 ± 28.2	155.3 ± 27.1	58.7 ± 12.9	151.7 ± 16.5	94.9 ± 32.6	137.4 ± 36.0
S-C-N(TS)	169.9 ± 6.0	171.5 ± 3.4	167.9 ± 5.0	171.3 ± 2.8	162.9 ± 19.1	171.8 ± 2.9

**Table 4:** Mean values for some selected interaction distances between the cofactor and substrate at the catalytic site, for both Y44F and Y128F mutants for their respective RS and TS for the di- and tri-methyl-transfer reactions. Distances are in Å and are provided with their corresponding standard deviations.

Systems	State	Y44-K4 ^a^	Y128-K4 ^a^	V68-Met ^b^	R89-Met ^b^	I91-Met ^b^	Y130-Met ^c^	Y69-SAM ^d^
Y44F-diMet	RS	---	2.36 ± 0.27	2.57 ± 0.25	2.55 ± 0.74	2.56 ± 0.88	2.55 ± 0.78	2.55 ± 1.19
	TS	---	2.45 ± 0.25	2.43 ± 0.12	2.40 ± 0.67	2.40 ± 0.40	2.39 ± 1.09	2.39 ± 1.23
Y44A-diMet	RS	---	2.65 ± 0.36	2.25 ± 1.42	3.17 ± 0.33	2.66 ± 0.69	2.95 ± 0.79	2.18 ± 1.27
	TS	---	2.71 ± 0.31	2.51 ± 0.13	3.22 ± 0.56	2.74 ± 0.43	2.72 ± 0.24	2.25 ± 1.42
Y128F-diMet	RS	2.79 ± 0.37	---	2.59 ± 0.42	3.32 ± 0.63	4.04 ± 0.39	2.63 ± 0.50	2.79 ± 0.59
	TS	2.57 ± 0.20	---	2.57 ± 0.40	3.47 ± 0.60	3.83 ± 0.44	3.06 ± 0.72	2.97 ± 0.71
Y128A-diMet	RS	2.29 ± 0.12	---	2.63 ± 0.26	2.95 ± 0.43	3.55 ± 0.51	3.31 ± 0.73	1.64 ± 0.21
	TS	2.57 ± 0.29	---	2.64 ± 0.38	2.79 ± 0.28	3.47 ± 0.46	2.90 ± 0.39	1.63 ± 0.21
Y44A-triMet	RS	---	3.94 ± 0.75	2.38 ± 0.10	3.77 ± 0.98	4.80 ± 1.68	5.18 ± 1.16	3.21 ± 1.32
	TS	---	4.03 ± 0.87	2.45 ± 0.18	3.65 ± 0.95	3.43 ± 0.67	3.36 ± 0.84	3.25 ± 1.32
Y128A-triMet	RS	2.61 ± 0.17	---	2.61 ± 0.29	3.27 ± 0.58	3.15 ± 1.13	4.32 ± 1.17	2.97 ± 1.56
	TS	2.60 ± 0.17	---	2.56 ± 0.36	3.57 ± 0.61	2.60 ± 0.31	3.31 ± 0.65	3.00 ± 1.44

^a^ Distance between the hydroxyl oxygen from Tyr and the closest proton from Lys4. ^b^ Distance between the carboxyl oxygen from the backbone of the residue and the closest hydrogen from Met. ^c^ Distance between the hydroxyl oxygen from Tyr and the closest hydrogen from Met. ^d^ Distance between the hydroxyl group from Tyr69 and the closest oxygen from the carboxylic acid of SAM. --- means no data in the column in the table.

**Table 5:** Values represent the percentage (%) of systems from the set of 25 conformers of each mutant state where Nε(K4) from the lysine interacts with a water molecule at the RS and TS.

	Mono-Methyl Transfer			
System	Y44F	Y44A	Y128F	Y128A
RS	84	52	84	0
TS	12	0	0	0
	**di-methyl transfer**			
	Y44F	Y44A	Y128F	Y128A
RS	72	16	60	8
TS	4	8	4	0
	**tri-methyl transfer**			
	Y128A	Y44A		
RS	0	88		
TS	0	0		

## Data Availability

The data presented in this study are available on request from the corresponding author.

## Supplementary Materials

The following supporting information can be downloaded at: https://www.mdpi.com/article/10.3390/ijms231810339/s1.
