# Supplementary material for: Unraveling the Role of the Tyrosine Tetrad from the Binding Site of the Epigenetic Writer MLL3 in the Catalytic Mechanism and Methylation Multiplicity
Source: Int J Mol Sci. 2022 Sep 7;23(18):10339. doi: 10.3390/ijms231810339 (PMC9499395; doi:10.3390/ijms231810339)
Supplement: Supplementary file 1 [file ijms-23-10339-s001.zip › ijms-1873739-supplementary.pdf]

Supplementary Material for:

Unraveling the role of the tyrosine-quartet from MLL3 biding site in the catalytic mechanism and methylation multiplicity

Kevin Blanco-Esperguez[1]. *Iñaki Tuñón*[2]. *Johannes Kästner*[3]. *Fernando Mendizábal*[4] *and Sebastián Miranda-Rojas*[1]*

[1]Departamento de Ciencias Químicas. Facultad de Ciencias Exactas. Universidad Andres Bello. República 275. Santiago. Chile.

[2]Departamento de Química Física. Universidad de Valencia. 46100 Burjasot (Spain)

[3]Institut für Theoretische Chemie. Universität Stuttgart. Pfaffenwaldring 55. 70569 Stuttgart. Germany.

[4]Departamento de Química. Facultad de Ciencias. Universidad de Chile. P.O. Box 653. Las Palmeras 3425. Ñuñoa. Santiago. Chile.

*Correspondence: sebastian.miranda@unab.cl (S.M-R)

1. Section S1……………………………………………………………...3
2. Figure S1……………………………………………………………...4
3. Section S2……………………………………………………………...5
4. Figure S2……………………………………………………………7
5. Figure S3……………………………………………………………8
6. Table S1……………………………………………………………9
7. Table S2……………………………………………………………10
8. Table S3……………………………………………………………11
9. Table S4……………………………………………………………12
10. Table S5……………………………………………………………13
11. Table S6……………………………………………………………14
12. Table S7……………………………………………………………15
13. Table S8……………………………………………………………16
14. Table S9……………………………………………………………17
15. Table S10……………………………………………………………...18
16. Table S11……………………………………………………………..........19
17. Table S12……………………………………………………………20
18. Table S13……………………………………………………………21
19. References……..……………………………………………………22

Section S1. Analysis of the viability for the lysine substrate deprotonation process in the mutants

In a previous study,[1] we performed a detailed study of the deprotonation mechanism of the lysine substrate carried out by MLL3. We found that the deprotonation step has an associated energy barrier smaller than that corresponding to the methylation step, which is the rate-limiting step of the process. In this context, two of the main factors were found to be determinant for the deprotonation: (i) the formation of a chain of molecules connecting the protonated lysine with the outside of the protein, and (ii) the presence of the SAM cofactor. The relevance of the SAM cofactor relies on its positive charge that makes the deprotonation process kinetically viable, probably by raising the energy of the protonated lysine by electrostatic repulsive effects. After the deprotonation step, the removal of the electrostatic repulsion between SAM and the lysine induces a reorganization of the catalytic site, excluding water molecules to facilitate the approach of the methyl group from SAM to the amine group of the lysine compacting the catalytic site. In the present article, we were able to identify that Y130 is the residue in charge of the main reorganization of the catalytic site and the main responsible of the water exclusion.

Regarding the effect of mutations on the deprotonation step, we have found that for the inactive mutants there was always chains of water molecules that could help to deprotonate the lysine, suggesting that for these cases, the deprotonation could take place without a significant change with respect to the wild type enzyme. On the other hand, for the cases of active mutants, it is expected that the exclusion of water molecules from the active site process takes place after the deprotonation, because the electrostatic repulsion between the positively charge lysine and SAM keep the catalytic site open to the environment, which suggests that the main variables needed for the deprotonation process should be still available for the mutants. To test this hypothesis, we

proceeded by running a 10 ns MD of the Y128A mutant with a protonated di-methylated lysine, as this system in its deprotonated state was exceedingly efficient in the water occlusion process. First, as expected, because of the electrostatic repulsion between the lysine and SAM, the catalytic site is open, allowing the access of several water molecules (see Figure S1). Then, we selected a proper snapshot according to the hydrogen bond network necessary for the deprotonation of the substrate and the transfer of the proton to the bulk and we were able to find a TS for this process. As expected, the energy barrier obtained, was of 11.8 kcal/mol making the process viable for this system, value that is in line with the energy barriers found for the same process in the native enzyme;[1] and thereby, confirming our initial assumptions that as long as the system keep the factors needed for the deprotonation process (presence of SAM and water molecules), the process will take place with an energy barrier smaller than for the methylation process.

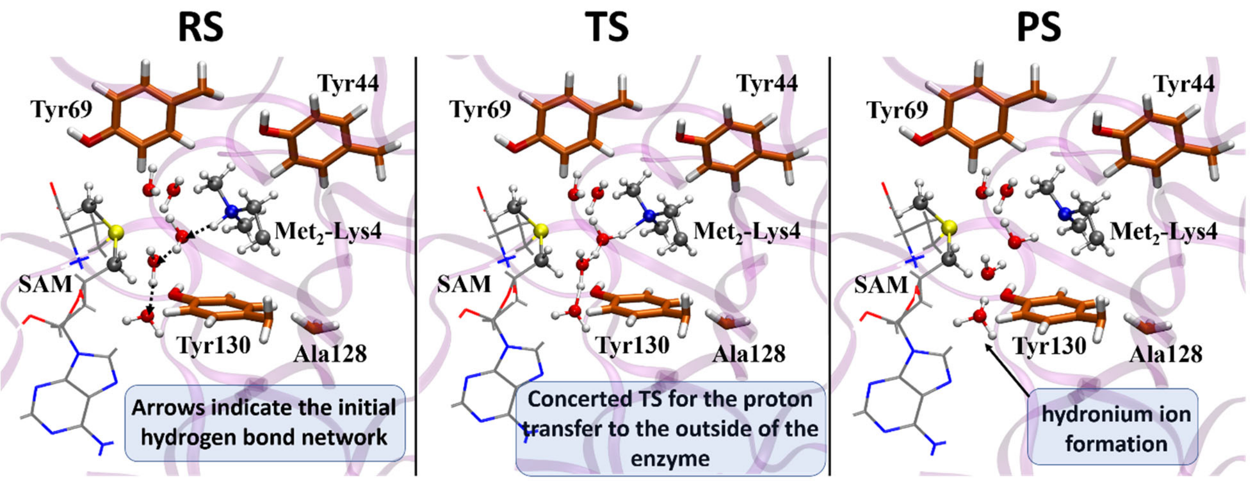

Figure S1. Reactant (RS), transition state (TS) and product (PS) for the deprotonation process of the lysine substrate carried out on the Y128A mutant with the lysine in its di-methylated form. Only a few water molecules are shown for the sake of clarity. While in the RS there is a clear formation of a hydrogen bond network, the most interesting part comes from the highly concerted nature of the TS where the proton is taken outside of the enzymatic environment in the form of a hydronium ion (enzyme's carbon—orange; substrate and cofactor carbon—gray; red—oxygen; blue—nitrogen; white—hydrogen).

Section 2. Correlation Analysis between $\Delta E^{\neq}$ and the distance travelled by the Methyl group during the transfer.

As previously pointed out for the native enzyme, the activation energy $\Delta E^{\neq}$ is linearly correlated to the distance traveled by the methyl group that is transferred. This magnitude d(ΔC···N) is obtained from the difference of the mean distances at the reactants and transition structures [d(C···N)RS – d(C···N)TS]. Figure S1 shows the plot and correlation factor (*R*) between $\Delta E^{\neq}$ and ΔC···N for the mono-methyl transfer of Y44F, Y128F, Y44A, Y128A, Y69F and Y130F. The value obtained of *R* for Y44F was of 0.604 and of 0.858 for Y128F. The low correlation factor obtained for Y44F implies that there are other factors besides the structural work that determine the energetic barrier. Meanwhile, the removal of one conformer that was outside of the trend ($M_{(128)}$-conf20 according to table S1) from the Y128F set, leads to an increase of the *R* value to 0.921; thus, showing that Y128F keeps the linear correlation between the structural work needed for the displacement of the methyl group necessary for the methylation process. Unexpectedly. the mutants Y44A and Y128A showed the opposite behavior, where Y44A shows a linear correlation between the $\Delta E^{\neq}$ with a *R* value of 0.944 if we removed only two conformations that came from the first 4 ns of simulation that clearly were outside of the trend. On the other hand. Y128A showed a *R* value of 0.618 denoting the lack of linear correlation between $\Delta E^{\neq}$ and ΔC···N; thus, indicating that in this case other factors are involved in the control of the barrier height. One of the possible factors affecting the correlation between the ΔC···N distance and $\Delta E^{\neq}$ for this S_N2 reaction could be exposed by the angle between the atoms from the reaction axis attributed to S···C···N, which should be close to 180° at the TS due to the type of orbital interaction needed for the reaction. Therefore. to minimize the structural work invested in reaching this target angle. this is expected

to be as close as possible to 180 at the RS. However, the angle values of 136.0° and 123.8° obtained for Y44F and 128F were in general lower and less favorable than the observed for the native enzyme (151.0°). This clearly shows a lower and less favorable angle for the S_N2 attack for the mutants. The main reason for low angles of attack is related to the incorporation of water molecules to the binding site, which as explained in the main article, is one of the main determinants of the deactivation of the enzyme and probably for the loss of the proper conformation of the catalytic machinery.

The plot of the ΔC···N distances against the $\Delta E^{\neq}$ for Y69F presented in Figure S1 shows a correlation factor (*R*) of 0.850, which denotes that there is a major effect in the positioning of the methyl group, which is reflected in an almost linear increase of $\Delta E^{\neq}$. As expected, there was an important increase in the mean C···N distance with a mean value of 4.01 Å, much higher than the native system (3.19 Å), and consequently leading to the increase of $\Delta E^{\neq}$. For Y130F the correlation plot showed a *R* value of 0.454, which on the other hand, indicates that this mutant impairs the reaction by a different mechanism than the regulation of the distance travelled by the methyl group. In this sense, apparently an important factor is the proper interaction pattern between the K4 and Y44/Y128, where the latter have to act as proton acceptors and thus, induce an adequate orientation of the K4 substrate and avoid the inactivation of the electron pair from Nε(K4). In this case, as exposed above, the entering of water molecules causes a complete change in the interaction configuration of the binding site, leading to the low correlation factor observed.

For the di- and tri-methylation process, the ΔC···N correlation with the $\Delta E^{\neq}$ is shown in Figure S2, where we found that for the Y44F and Y128F there is a high dispersion of the data without showing a linear correlation. Meanwhile, for Y44A and Y128A mutants, there is some degree of linear correlation with *R* values of 0.886 and 0.842; respectively. Actually, when for Y128A we

removed one snapshot from the data set that corresponded to a conformation from the beginning of the simulation, the *R* value is increased to 0.973 showing a high linear correlation between the distances travelled by the methyl group and the barrier height. Clearly, for the most active forms of the enzyme, in which there are no water molecules in the binding site, the correlation factor increases. According to figure S2e and S2f, the correlation between $\Delta E^{\neq}$ and $\Delta C \cdots N$ seems to be low with only a slight correlation between these two parameters and *R* values of 0.572 and 0.644, but in this case is probably due to a highly crowded catalytic site.

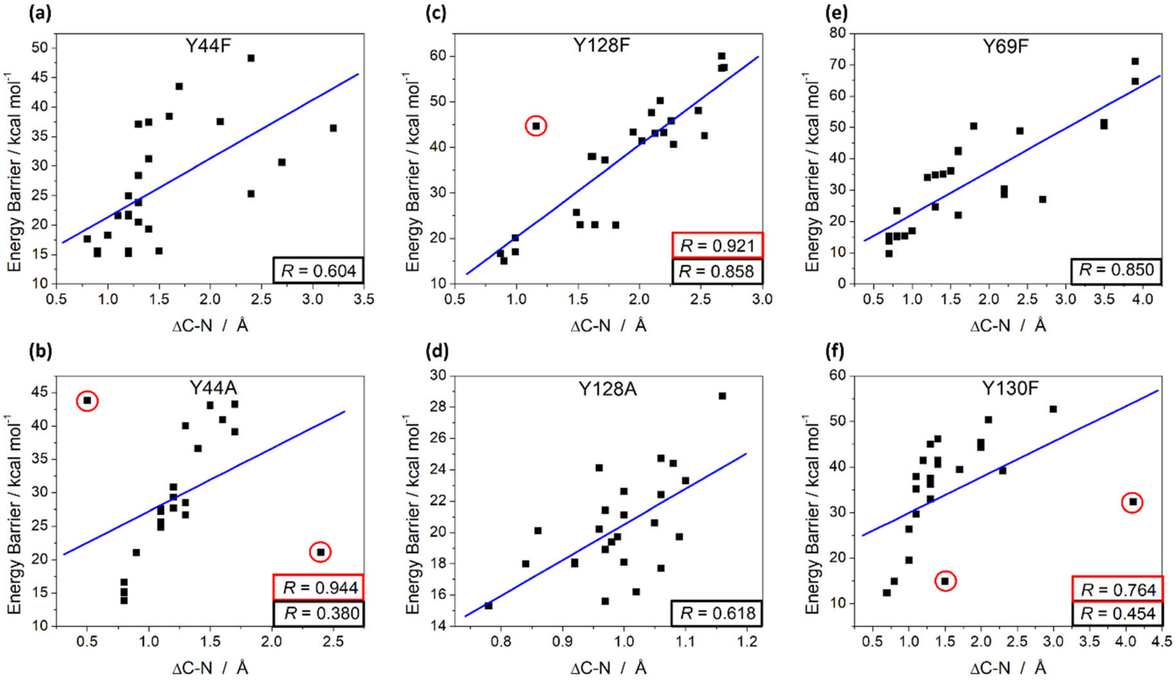

Figure S2. Plots of the barrier height ($\Delta E^{\neq}$) for the first methyl transfer reaction versus the distance traveled by the Met group along the reaction coordinate to form mono-methyl lysine. Framed in black is the correlation factor including the complete series, in red is the correlation factor removing the data out of the trend which are circled in red.

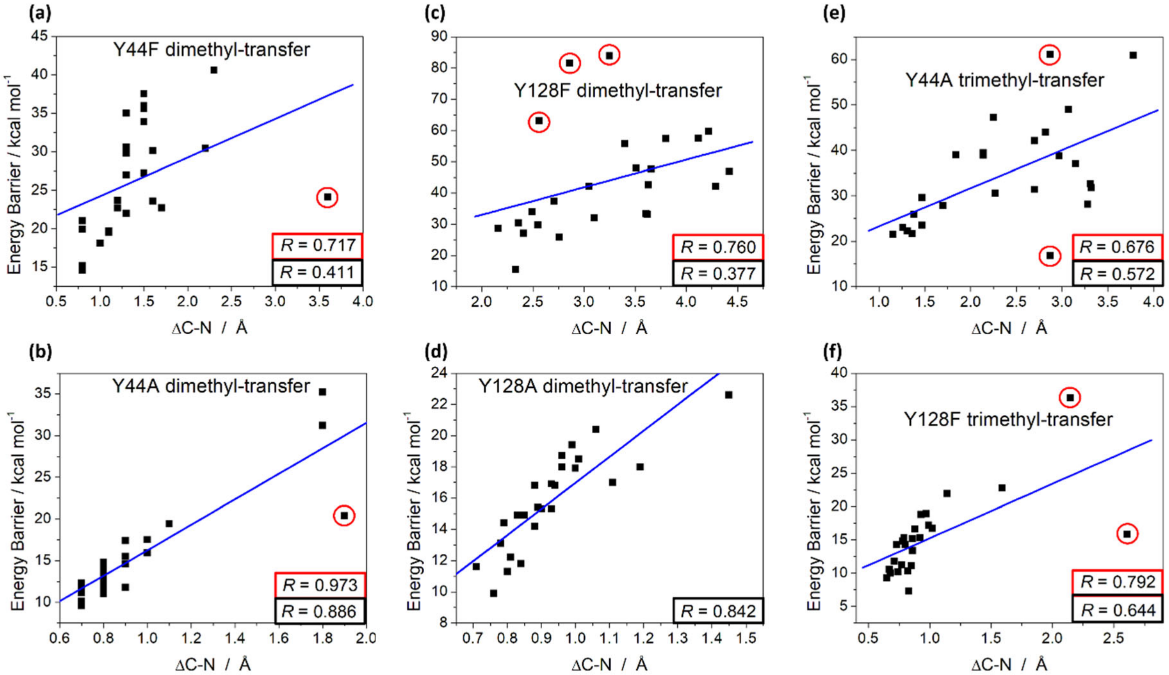

Figure S3. Plots of the barrier height ($\Delta E^{\neq}$) for the second and third methyl transfer reaction versus the distance traveled by the Met group along the reaction coordinate to form di- and tri-methyl lysine. Framed in black is the correlation factor including the complete series, in red is the correlation factor removing the data out of the trend which are circled in red.

Table S1. Correlation between the numbering used in this article for the MLL3 subunit and the ones used in the article from which the crystal structure was obtained published by *Li et al.*[2]

Crystal structure article	This article
Tyr4800	Tyr44
Tyr4825	Tyr69
Tyr4884	Tyr128
Tyr4886	Tyr130

Table S2. Selected geometric parameters for the conformations at the RS and TS for the Y128F methyl transfer reaction carried out by the mutated M3RA-Y128F. Energies are in kcal/mol. distances in Å and angles in degrees (°)

System	ΔE≠ ± S	$\Delta E_2°$ ± S	*C-N(RS) ± S*	*C-N(TS) ± S*	*S-C(RS) ± S*	*S-C(TS) ± S*	*S-C-N(RS) ± S*	*S-C-N(TS) ± S*
$M_{(128)}$-conf-1	20.1	-15.3	3.20	2.21	1.81	2.28	175.5	176.9
$M_{(128)}$-conf-2	17.1	-11.1	3.15	2.16	1.81	2.26	139.6	168.8
$M_{(128)}$-conf-3	15.0	-15.9	3.08	2.18	1.82	2.25	148.5	166.5
$M_{(128)}$-conf-4	16.7	-16.3	3.10	2.23	1.80	2.24	126.5	165.8
$M_{(128)}$-conf-5	23.0	-12.3	3.74	2.22	1.81	2.30	122.1	171.0
$M_{(128)}$-conf-6	22.9	-8.5	4.01	2.20	1.81	2.28	149.6	165.0
$M_{(128)}$-conf-7	23.0	-5.2	3.83	2.19	1.82	2.31	132.6	164.3
$M_{(128)}$-conf-8	25.7	-4.2	3.73	2.24	1.81	2.31	128.3	171.0
$M_{(128)}$-conf-9	37.2	-4.4	4.06	2.34	1.81	2.33	119.3	152.3
$M_{(128)}$-conf-10	45.7	-11.9	4.75	2.49	1.81	2.40	109.1	158.3
$M_{(128)}$-conf-11	40.6	-11.2	4.72	2.44	1.81	2.28	105.2	159.8
$M_{(128)}$-conf-12	57.5	-3.6	5.23	2.54	1.82	2.47	110.2	155.6
$M_{(128)}$-conf-13	60.0	2.8	5.24	2.57	1.81	2.51	120.6	158.6
$M_{(128)}$-conf-14	41.4	-3.7	4.42	2.40	1.81	2.37	110.3	152.5
$M_{(128)}$-conf-15	48.0	0.4	4.93	2.45	1.81	2.42	120.9	152.1
$M_{(128)}$-conf-16	43.2	-10.6	4.72	2.52	1.81	2.44	116.4	157.1
$M_{(128)}$-conf-17	43.3	1.1	4.38	2.43	1.80	2.38	110.1	161.9
$M_{(128)}$-conf-18	37.9	-3.8	4.15	2.54	1.81	2.32	115.1	146.9
$M_{(128)}$-conf-19	42.5	-6.2	5.08	2.55	1.81	2.50	120.0	150.8
$M_{(128)}$-conf-20	44.6	-2.5	3.74	2.58	1.81	2.78	96.1	109.5
$M_{(128)}$-conf-21	57.3	15.5	5.12	2.45	1.81	2.51	113.2	154.6
$M_{(128)}$-conf-22	50.2	1.1	4.60	2.43	1.81	2.42	109.0	163.0
$M_{(128)}$-conf-23	47.6	0.3	4.53	2.43	1.81	2.45	117.7	164.6
$M_{(128)}$-conf-24	38.0	-2.0	4.01	2.39	1.81	2.36	113.7	164.9
$M_{(128)}$-conf-25	43.0	-9.9	4.66	2.53	1.80	2.37	109.7	157.1
Avg. (Ā)	37.7 ± 13.5	-7.1 ± 7.2	4.16 ± 0.68	2.37 ± 0.14	1.81 ± 0.01	2.37 ± 0.12	123.8 ± 17.0	158.2 ± 12.6

Table S3. Selected geometric parameters for the conformations at the RS and TS for the Y44F methyl transfer reaction carried out by the mutated M3RA-Y44F. Energies are in kcal/mol. distances in Å and angles in degrees (°)

System	ΔE≠ ± S	$\Delta E_2°$ ± S	*C-N(RS) ± S*	*C-N(TS) ± S*	*S-C(RS) ± S*	*S-C(TS) ± S*	*S-C-N(RS) ± S*	*S-C-N(TS) ± S*
$M_{(44)}$-conf-1	15.1	-18.2	3.14	2.25	1.81	2.27	131.8	170.8
$M_{(44)}$-conf-2	25.0	-7.5	3.42	2.21	1.82	2.29	114.0	162.9
$M_{(44)}$-conf-3	25.3	-10.5	4.64	2.28	1.82	2.22	134.9	166.8
$M_{(44)}$-conf-4	18.3	-20.9	3.28	2.30	1.81	2.24	138.4	164.7
$M_{(44)}$-conf-5	21.8	-13.3	3.41	2.23	1.81	2.23	138.9	175.0
$M_{(44)}$-conf-6	20.5	-11.6	3.52	2.21	1.82	2.18	103.7	166.6
$M_{(44)}$-conf-7	15.2	-26.2	3.42	2.26	1.81	2.17	156.0	168.0
$M_{(44)}$-conf-8	15.6	-23.9	3.23	2.31	1.82	2.21	146.6	174.1
$M_{(44)}$-conf-9	15.6	-22.3	3.73	2.26	1.82	2.23	110.4	166.3
$M_{(44)}$-conf-10	31.2	-10.8	3.58	2.20	1.82	2.44	126.6	162.0
$M_{(44)}$-conf-11	15.6	-28.3	3.55	2.32	1.82	2.29	146.5	165.0
$M_{(44)}$-conf-12	17.6	-30.0	3.22	2.38	1.82	2.38	156.1	172.7
$M_{(44)}$-conf-13	37.4	-16.8	3.70	2.33	1.82	2.44	123.7	167.7
$M_{(44)}$-conf-14	36.4	-1.8	5.55	2.40	1.82	2.47	162.3	164.4
$M_{(44)}$-conf-15	30.6	-21.9	5.09	2.44	1.82	2.37	113.3	162.4
$M_{(44)}$-conf-16	43.5	-7.1	3.87	2.21	1.82	2.52	109.9	156.3
$M_{(44)}$-conf-17	37.5	-20.4	4.42	2.31	1.82	2.45	149.2	164.8
$M_{(44)}$-conf-18	12.3	-18.3	3.18	2.23	1.82	2.27	143.3	171.7
$M_{(44)}$-conf-19	28.4	-14.2	3.61	2.31	1.82	2.34	154.8	171.3
$M_{(44)}$-conf-20	38.4	-9.2	3.88	2.26	1.82	2.48	122.2	154.2
$M_{(44)}$-conf-21	37.1	-9.0	3.65	2.34	1.82	2.38	126.4	169.0
$M_{(44)}$-conf-22	48.3	7.6	4.61	2.26	1.81	2.42	112.7	163.0
$M_{(44)}$-conf-23	21.6	-8.7	3.33	2.22	1.81	2.38	133.4	162.6
$M_{(44)}$-conf-24	19.3	-10.4	3.55	2.20	1.81	2.27	155.8	173.4
$M_{(44)}$-conf-25	23.8	-10.8	3.52	2.26	1.82	2.28	166.8	172.1
Avg. (Ā)	26.4 ± 9.9	-14.2 ± 8.7	3.77 ± 0.61	2.28 ± 0.06	1.82 ± 0.00	2.34 ± 0.10	136.0 ± 19.4	166.7 ± 5.3

Table S4. Selected geometric parameters for the conformations at the RS and TS for the Y69F methyl transfer reaction carried out by the mutated M3RA-Y69F. Energies are in kcal/mol. distances in Å and angles in degrees (°)

System	ΔE≠ ± S	$\Delta E_2°$ ± S	*C-N(RS) ± S*	*C-N(TS) ± S*	*S-C(RS) ± S*	*S-C(TS) ± S*	*S-C-N(RS) ± S*	*S-C-N(TS) ± S*
$M_{(69)}$-conf-1	15.4	-14.2	2.99	2.17	1.82	2.35	153.6	167.2
$M_{(69)}$-conf-2	9.6	-22.3	2.93	2.22	1.83	2.27	167.2	171.4
$M_{(69)}$-conf-3	42.6	7.4	3.85	2.28	1.82	2.46	134.2	169.8
$M_{(69)}$-conf-4	34.8	2.6	3.48	2.19	1.82	2.54	151.9	168.5
$M_{(69)}$-conf-5	34.0	4.6	3.35	2.17	1.81	2.48	150.0	167.2
$M_{(69)}$-conf-6	35.0	-8.2	3.59	2.22	1.81	2.39	152.6	157.2
$M_{(69)}$-conf-7	50.3	15.3	3.98	2.23	1.82	2.80	157.4	168.5
$M_{(69)}$-conf-8	42.2	-7.9	3.85	2.21	1.82	2.65	149.2	165.8
$M_{(69)}$-conf-9	15.4	-10.4	3.08	2.18	1.81	2.31	166.3	177.3
$M_{(69)}$-conf-10	30.4	4.2	4.44	2.26	1.82	2.47	152.1	178.6
$M_{(69)}$-conf-11	27.0	-6.1	4.98	2.27	1.82	2.36	168.2	170.5
$M_{(69)}$-conf-12	36.1	0.4	3.74	2.29	1.82	2.47	145.5	166.5
$M_{(69)}$-conf-13	15.1	-12.5	2.91	2.16	1.80	2.25	151.0	167.3
$M_{(69)}$-conf-14	23.3	-9.6	3.10	2.28	1.81	2.36	139.2	172.2
$M_{(69)}$-conf-15	15.3	-10.9	2.96	2.22	1.82	2.31	166.7	175.1
$M_{(69)}$-conf-16	16.9	-12.0	3.22	2.26	1.82	2.33	154.8	171.5
$M_{(69)}$-conf-17	24.6	-16.6	3.53	2.27	1.82	2.27	113.9	162.4
$M_{(69)}$-conf-18	50.4	8.9	5.78	2.24	1.81	2.63	140.5	164.6
$M_{(69)}$-conf-19	64.7	-11.3	6.47	2.57	1.81	2.69	129.7	153.7
$M_{(69)}$-conf-20	48.8	-6.9	4.63	2.22	1.82	2.61	118.7	160.1
$M_{(69)}$-conf-21	13.7	-12.8	2.90	2.20	1.82	2.28	170.8	175.3
$M_{(69)}$-conf-22	28.5	-4.2	4.48	2.24	1.82	2.36	101.5	163.4
$M_{(69)}$-conf-23	21.9	-15.2	3.78	2.19	1.82	2.30	114.4	165.1
$M_{(69)}$-conf-24	51.4	-5.2	5.74	2.29	1.81	2.53	122.0	176.9
$M_{(69)}$-conf-25	71.0	9.2	6.48	2.54	1.82	2.51	135.0	162.6
Avg. (Ā)	32.7 ± 16.6	-5.4 ± 9.5	4.01 ± 1.11	2.25 ± 0.10	1.82 ± 0.01	2.44 ± 0.15	144.2 ± 19.0	167.9 ± 6.2

Table S5. Selected geometric parameters for the conformations at the RS and TS for the Y130F methyl transfer reaction carried out by the mutated M3RA-Y69F. Energies are in kcal/mol. distances in Å and angles in degrees (°)

System	ΔE≠ ± S	ΔE_2° ± S	*C-N(RS) ± S*	*C-N(TS) ± S*	*S-C(RS) ± S*	*S-C(TS) ± S*	*S-C-N(RS) ± S*	*S-C-N(TS) ± S*
$M_{(130)}$-conf-1	20.0	-12.7	3.05	2.23	1.80	2.25	117.3	161.4
$M_{(130)}$-conf-2	32.3	-10.7	6.37	2.27	1.82	2.24	50.8	164.9
$M_{(130)}$-conf-3	14.9	-20.4	3.84	2.31	1.81	2.26	75.6	168.9
$M_{(130)}$-conf-4	32.9	-7.1	3.58	2.26	1.81	2.19	104.5	164.8
$M_{(130)}$-conf-5	46.1	15.6	3.56	2.19	1.82	2.68	137.6	158.4
$M_{(130)}$-conf-6	37.8	-2.3	3.30	2.17	1.82	2.50	128.9	163.7
$M_{(130)}$-conf-7	45.3	14.9	4.17	2.21	1.82	2.66	134.3	163.9
$M_{(130)}$-conf-8	44.3	2.9	4.19	2.15	1.81	2.46	77.4	171.8
$M_{(130)}$-conf-9	39.1	-10.2	4.59	2.32	1.82	2.30	65.6	170.6
$M_{(130)}$-conf-10	52.6	0.7	5.36	2.37	1.82	2.35	51.4	155.0
$M_{(130)}$-conf-11	39.4	-9.0	3.89	2.23	1.81	2.36	86.0	173.5
$M_{(130)}$-conf-12	44.9	-0.4	3.42	2.11	1.81	2.45	114.3	144.0
$M_{(130)}$-conf-13	35.1	-12.0	3.18	2.09	1.81	2.38	134.3	153.7
$M_{(130)}$-conf-14	50.3	-10.1	4.37	2.29	1.81	2.29	86.2	161.6
$M_{(130)}$-conf-15	41.4	5.2	3.59	2.41	1.81	2.18	118.8	159.3
$M_{(130)}$-conf-16	36.1	-1.0	3.47	2.18	1.80	2.34	117.3	153.7
$M_{(130)}$-conf-17	19.5	-13.1	3.30	2.27	1.81	2.32	160.4	176.5
$M_{(130)}$-conf-18	40.5	6.0	3.53	2.18	1.82	2.46	136.2	156.4
$M_{(130)}$-conf-19	41.4	-1.9	3.56	2.21	1.81	2.40	116.4	165.8
$M_{(130)}$-conf-20	12.3	-19.5	2.92	2.25	1.83	2.27	169.2	170.6
$M_{(130)}$-conf-21	35.1	-8.5	3.34	2.24	1.80	2.40	129.1	159.4
$M_{(130)}$-conf-22	29.6	-18.0	3.35	2.23	1.81	2.37	132.6	166.1
$M_{(130)}$-conf-23	14.9	-12.8	3.02	2.23	1.82	2.31	168.5	175.3
$M_{(130)}$-conf-24	37.4	-4.9	3.48	2.23	1.81	2.41	125.1	160.5
$M_{(130)}$-conf-25	26.3	-2.4	3.16	2.21	1.81	2.23	165.9	175.2
Avg. (Ā)	34.8 ± 11.2	-5.3 ± 9.5	3.74 ± 0.78	2.23 ± 0.07	1.81 ± 0.01	2.36 ± 0.13	116.2 ± 34.3	163.8 ± 8.0

Table S6. Selected geometric parameters for the conformations at the RS and TS for the Y128A methyl transfer reaction carried out by the mutated M3RA-Y128A. Energies are in kcal/mol. distances in Å and angles in degrees (°)

System	ΔE≠ ± S	$ΔE_2$° ± S	*C-N(RS) ± S*	*C-N(TS) ± S*	*S-C(RS) ± S*	*S-C(TS) ± S*	*S-C-N(RS) ± S*	*S-C-N(TS) ± S*
$M_{(128)}$-conf-1	19.7	-6.5	3.15	2.16	1.81	2.32	124.9	167.7
$M_{(128)}$-conf-2	18.1	-11.8	3.09	2.17	1.81	2.30	121.1	168.5
$M_{(128)}$-conf-3	20.6	-1.5	3.20	2.15	1.82	2.37	148.1	172.9
$M_{(128)}$-conf-4	17.7	-14.5	3.30	2.24	1.82	2.34	139.1	173.3
$M_{(128)}$-conf-5	18.0	-15.7	3.08	2.24	1.81	1.82	121.0	163.4
$M_{(128)}$-conf-6	21.1	-9.9	3.25	2.25	1.81	2.31	133.6	168.7
$M_{(128)}$-conf-7	15.3	-9.2	2.97	2.19	1.83	2.33	174.5	177.2
$M_{(128)}$-conf-8	19.7	-11.7	3.34	2.25	1.81	2.29	135.0	169.5
$M_{(128)}$-conf-9	18.1	-11.1	3.23	2.23	1.81	2.29	142.2	170.4
$M_{(128)}$-conf-10	18.9	-15.5	3.28	2.31	1.81	2.27	133.3	169.6
$M_{(128)}$-conf-11	18.0	-7.8	3.11	2.19	1.81	2.30	151.2	169.6
$M_{(128)}$-conf-12	24.1	-8.3	3.19	2.23	1.82	2.17	93.2	170.3
$M_{(128)}$-conf-13	20.1	-10.4	3.05	2.19	1.81	2.26	109.4	161.6
$M_{(128)}$-conf-14	19.7	-2.8	3.15	2.16	1.82	2.32	119.2	174.1
$M_{(128)}$-conf-15	23.3	-2.0	3.28	2.18	1.81	2.37	126.3	172.0
$M_{(128)}$-conf-16	28.7	-8.1	3.48	2.25	1.80	2.36	144.3	165.9
$M_{(128)}$-conf-17	19.4	-4.7	3.17	2.19	1.81	2.34	133.7	169.6
$M_{(128)}$-conf-18	24.4	3.0	3.22	2.14	1.81	2.36	128.0	167.2
$M_{(128)}$-conf-19	20.2	-1.9	3.10	2.14	1.82	2.37	165.6	173.8
$M_{(128)}$-conf-20	24.7	-2.3	3.28	2.22	1.81	2.36	124.6	168.1
$M_{(128)}$-conf-21	15.6	-9.4	3.10	2.13	1.80	2.28	126.4	165.4
$M_{(128)}$-conf-22	22.4	-2.8	3.26	2.20	1.81	2.36	123.3	170.9
$M_{(128)}$-conf-23	21.4	-5.7	3.17	2.20	1.81	2.31	127.4	169.9
$M_{(128)}$-conf-24	22.6	-2.8	3.19	2.19	1.81	2.32	127.7	169.2
$M_{(128)}$-conf-25	16.2	-14.5	3.31	2.29	1.82	2.32	147.5	174.1
Avg. (Ā)	20.3 ± 3.1	-7.5 ± 5.1	3.20 ± 0.10	2.20 ± 0.05	1.81 ± 0.01	2.30 ± 0.11	132.8 ± 16.7	169.7 ± 3.5

Table S7. Selected geometric parameters for the conformations at the RS and TS for the Y44A methyl transfer reaction carried out by the mutated M3RA-Y44A. Energies are in kcal/mol. distances in Å and angles in degrees (°)

System	ΔE≠ ± S	$\Delta E_2°$ ± S	*C-N(RS) ± S*	*C-N(TS) ± S*	*S-C(RS) ± S*	*S-C(TS) ± S*	*S-C-N(RS) ± S*	*S-C-N(TS) ± S*
$M_{(44)}$-conf-1	21.1	-11.0	4.65	2.21	1.81	2.25	47.7	167.7
$M_{(44)}$-conf-2	43.8	-25.8	2.98	2.44	1.81	2.62	85.5	75.8
$M_{(44)}$-conf-3	27.7	-7.1	3.50	2.29	1.81	2.27	99.4	160.8
$M_{(44)}$-conf-4	27.3	31.3	3.36	2.27	1.81	2.28	104.1	159.1
$M_{(44)}$-conf-5	27.2	-4.1	3.37	2.26	1.81	2.26	113.3	165.9
$M_{(44)}$-conf-6	15.0	-11.7	2.99	2.19	1.83	2.28	170.2	173.4
$M_{(44)}$-conf-7	30.8	-4.2	3.28	2.13	1.81	2.36	124.2	166.2
$M_{(44)}$-conf-8	13.8	-15.2	3.03	2.19	1.88	2.29	147.7	171.2
$M_{(44)}$-conf-9	29.3	-5.9	3.42	2.22	1.81	2.38	129.0	166.4
$M_{(44)}$-conf-10	24.9	-7.7	3.31	2.20	1.81	2.33	124.3	165.9
$M_{(44)}$-conf-11	27.5	-9.4	3.30	2.25	1.80	2.26	113.8	165.3
$M_{(44)}$-conf-12	21.0	-6.8	3.17	2.23	1.81	2.26	124.4	169.3
$M_{(44)}$-conf-13	16.6	-14.3	2.93	2.12	1.82	2.28	170.7	172.5
$M_{(44)}$-conf-14	26.7	-6.0	3.42	2.17	1.81	2.35	122.0	166.2
$M_{(44)}$-conf-15	15.2	-13.5	2.96	2.18	1.82	2.26	170.8	172.7
$M_{(44)}$-conf-16	24.8	-6.3	3.32	2.20	1.81	2.38	131.1	168.4
$M_{(44)}$-conf-17	28.5	-5.5	3.42	2.14	1.81	2.42	128.1	163.9
$M_{(44)}$-conf-18	43.0	1.0	3.64	2.17	1.82	2.60	145.6	163.0
$M_{(44)}$-conf-19	39.1	-8.6	3.85	2.20	1.81	2.52	130.3	163.6
$M_{(44)}$-conf-20	43.2	6.3	3.93	2.23	1.82	2.58	115.0	149.9
$M_{(44)}$-conf-21	36.6	-6.1	3.59	2.20	1.81	2.62	140.9	160.0
$M_{(44)}$-conf-22	40.0	1.5	3.46	2.16	1.82	2.58	129.0	147.1
$M_{(44)}$-conf-23	25.6	-7.2	3.42	2.28	1.82	2.41	120.3	164.7
$M_{(44)}$-conf-24	40.9	0.2	3.81	2.26	1.82	2.65	133.4	165.0
$M_{(44)}$-conf-25	43.1	1.6	3.64	2.17	1.82	2.67	133.6	162.1
Avg. (Ā)	29.3 ± 9.6	-5.4 ± 10.1	3.43 ± 0.37	2.21 ± 0.07	1.82 ± 0.01	2.41 ± 0.15	126.2 ± 26.4	161.0 ± 18.8

Table S8. Selected geometric parameters for the conformations at the RS and TS for the Y128F methyl transfer reaction carried out by the mutated M3RA-Y128F. Energies are in kcal/mol. distances in Å and angles in degrees (°)

System	ΔE≠ ± S	$\Delta E_2°$ ± S	*C-N(RS) ± S*	*C-N(TS) ± S*	*S-C(RS) ± S*	*S-C(TS) ± S*	*S-C-N(RS) ± S*	*S-C-N(TS) ± S*
$M_{(128)}$-conf-1	15.5	-11.0	4.53	2.20	1.81	2.33	41.8	168.0
$M_{(128)}$-conf-2	33.3	-4.8	5.87	2.26	1.82	2.28	34.8	167.0
$M_{(128)}$-conf-3	42.1	1.9	6.57	2.28	1.82	2.42	43.7	173.3
$M_{(128)}$-conf-4	81.5	-2.3	5.10	2.24	1.82	2.34	53.0	167.5
$M_{(128)}$-conf-5	29.7	-5.4	4.87	2.32	1.82	2.35	52.8	164.8
$M_{(128)}$-conf-6	63.1	-15.1	4.80	2.24	1.81	2.17	52.5	172.2
$M_{(128)}$-conf-7	27.1	-8.3	4.68	2.27	1.82	2.14	56.0	168.0
$M_{(128)}$-conf-8	25.8	-4.2	4.97	2.21	1.81	2.21	51.7	171.4
$M_{(128)}$-conf-9	28.7	-3.6	4.35	2.19	1.81	2.19	60.2	163.9
$M_{(128)}$-conf-10	32.0	1.6	5.37	2.27	1.82	2.30	45.1	168.0
$M_{(128)}$-conf-11	30.4	-6.1	4.63	2.27	1.81	2.28	59.6	170.7
$M_{(128)}$-conf-12	33.1	-9.6	5.94	2.32	1.81	2.27	48.5	176.4
$M_{(128)}$-conf-13	42.6	-1.0	5.97	2.34	1.82	2.26	60.5	172.0
$M_{(128)}$-conf-14	55.7	0.3	5.91	2.51	1.81	2.26	77.7	153.5
$M_{(128)}$-conf-15	42.1	3.6	5.38	2.33	1.83	2.35	98.1	164.5
$M_{(128)}$-conf-16	57.5	4.4	6.52	2.40	1.82	2.46	58.7	164.2
$M_{(128)}$-conf-17	46.8	11.5	6.69	2.27	1.82	2.36	57.7	172.9
$M_{(128)}$-conf-18	57.3	11.3	6.17	2.37	1.81	2.30	64.0	168.1
$M_{(128)}$-conf-19	46.8	11.5	6.69	2.27	1.82	2.36	57.7	172.9
$M_{(128)}$-conf-20	59.7	9.0	6.46	2.24	1.81	2.40	63.6	169.5
$M_{(128)}$-conf-21	47.6	1.7	6.07	2.41	1.82	2.27	56.7	167.7
$M_{(128)}$-conf-22	83.9	26.6	5.57	2.32	1.81	2.53	66.2	159.5
$M_{(128)}$-conf-23	48.0	7.2	5.94	2.43	1.80	2.38	72.3	173.7
$M_{(128)}$-conf-24	37.4	-2.9	4.91	2.20	1.81	2.23	61.9	162.6
$M_{(128)}$-conf-25	34.0	-1.1	4.71	2.22	1.81	2.22	73.6	166.7
Avg. (Ā)	44.1 ± 16.7	0.6 ± 8.9	5.55 ± 0.75	2.30 ± 0.08	1.81 ± 0.01	2.31 ± 0.09	58.7 ± 12.9	167.9 ± 5.0

Table S9. Selected geometric parameters for the conformations at the RS and TS for the Y128A methyl transfer reaction carried out by the mutated M3RA-Y128A. Energies are in kcal/mol. distances in Å and angles in degrees (°)

System	ΔE≠ ± S	ΔE₂° ± S	*C-N(RS) ± S*	*C-N(TS) ± S*	*S-C(RS) ± S*	*S-C(TS) ± S*	*S-C-N(RS) ± S*	*S-C-N(TS) ± S*
$M_{(128)}$-conf-1	20.4	-7.7	3.28	2.22	1.81	2.34	134.5	167.6
$M_{(128)}$-conf-2	17.0	-6.0	3.23	2.12	1.82	2.30	113.3	169.5
$M_{(128)}$-conf-3	18.7	-16.2	3.25	2.29	1.81	2.31	130.7	166.3
$M_{(128)}$-conf-4	19.4	-8.5	3.20	2.21	1.81	2.31	139.8	171.1
$M_{(128)}$-conf-5	16.9	-12.7	3.18	2.25	1.82	2.32	154.0	172.5
$M_{(128)}$-conf-6	15.4	-15.3	3.13	2.24	1.82	2.32	158.4	169.4
$M_{(128)}$-conf-7	17.9	-12.2	3.24	2.24	1.82	2.36	160.7	172.2
$M_{(128)}$-conf-8	11.3	-16.0	3.00	2.20	1.82	2.24	163.5	172.8
$M_{(128)}$-conf-9	14.4	-11.5	2.94	2.15	1.82	2.27	157.0	167.9
$M_{(128)}$-conf-10	14.9	-10.2	3.02	2.17	1.82	2.30	163.2	169.6
$M_{(128)}$-conf-11	11.8	-14.9	3.01	2.17	1.82	2.20	158.6	173.9
$M_{(128)}$-conf-12	11.6	-18.3	2.95	2.24	1.83	2.27	169.9	174.0
$M_{(128)}$-conf-13	16.8	-9.6	3.07	2.19	1.82	2.33	163.2	170.1
$M_{(128)}$-conf-14	16.8	-4.2	3.06	2.12	1.82	2.32	159.9	171.3
$M_{(128)}$-conf-15	14.2	-14.4	3.09	2.21	1.82	2.29	157.3	170.8
$M_{(128)}$-conf-16	15.3	-11.0	3.08	2.18	1.82	2.29	154.4	169.4
$M_{(128)}$-conf-17	15.3	-9.4	3.10	2.17	1.82	2.31	176.2	179.1
$M_{(128)}$-conf-18	13.1	-20.0	3.06	2.28	1.83	2.30	167.4	172.7
$M_{(128)}$-conf-19	9.9	-19.2	2.97	2.21	1.82	2.23	171.1	176.1
$M_{(128)}$-conf-20	18.0	-9.8	3.15	2.19	1.81	2.32	152.9	172.4
$M_{(128)}$-conf-21	22.6	-8.8	3.69	2.24	1.81	2.33	134.4	170.2
$M_{(128)}$-conf-22	18.0	-16.6	3.47	2.28	1.80	2.25	126.6	169.6
$M_{(128)}$-conf-23	14.9	-19.6	3.07	2.24	1.80	2.22	129.0	169.0
$M_{(128)}$-conf-24	18.5	-9.0	3.19	2.18	1.81	2.31	133.4	172.1
$M_{(128)}$-conf-25	12.2	-22.3	3.04	2.23	1.82	2.24	163.1	173.9
Avg. (Ā)	15.8 ± 3.1	-12.9 ± 4.8	3.14 ± 0.17	2.21 ± 0.05	1.82 ± 0.01	2.29 ± 0.04	151.7 ± 16.5	171.3 ± 2.8

Table S10. Selected geometric parameters for the conformations at the RS and TS for the Y44F methyl transfer reaction carried out by the mutated M3RA-Y44F. Energies are in kcal/mol. distances in Å and angles in degrees (°)

System	ΔE≠ ± S	$\Delta E_2°$ ± S	*C-N(RS) ± S*	*C-N(TS) ± S*	*S-C(RS) ± S*	*S-C(TS) ± S*	*S-C-N(RS) ± S*	*S-C-N(TS) ± S*
$M_{(44)}$-conf-1	24.1	-9.8	5.87	2.25	1.82	2.22	46.9	175.4
$M_{(44)}$-conf-2	21.0	-16.9	3.09	2.25	1.81	2.18	109.2	162.4
$M_{(44)}$-conf-3	19.9	-17.3	3.08	2.28	1.81	2.20	107.0	160.2
$M_{(44)}$-conf-4	35.0	-6.0	3.56	2.23	1.81	2.30	120.4	163.1
$M_{(44)}$-conf-5	23.7	-10.5	3.40	2.24	1.81	2.19	115.1	163.7
$M_{(44)}$-conf-6	33.9	-3.9	3.83	2.34	1.81	2.30	112.7	166.3
$M_{(44)}$-conf-7	30.1	-18.6	3.93	2.34	1.81	2.24	104.1	168.6
$M_{(44)}$-conf-8	40.6	-4.3	4.66	2.37	1.81	2.27	104.2	153.0
$M_{(44)}$-conf-9	15.2	-15.9	3.08	2.27	1.81	2.31	149.4	172.1
$M_{(44)}$-conf-10	22.7	-8.8	3.97	2.30	1.81	2.35	157.7	172.8
$M_{(44)}$-conf-11	19.5	-10.3	3.38	2.25	1.81	2.36	139.6	176.1
$M_{(44)}$-conf-12	23.6	-4.3	3.79	2.23	1.81	2.29	138.5	172.1
$M_{(44)}$-conf-13	19.7	-9.6	3.38	2.24	1.82	2.38	166.3	174.9
$M_{(44)}$-conf-14	22.0	-12.5	3.49	2.24	1.81	2.36	156.3	176.1
$M_{(44)}$-conf-15	14.6	-16.2	3.00	2.23	1.83	2.34	171.7	172.8
$M_{(44)}$-conf-16	30.4	4.9	4.39	2.21	1.82	2.40	160.3	171.5
$M_{(44)}$-conf-17	27.2	-7.6	3.75	2.28	1.82	2.37	165.7	175.3
$M_{(44)}$-conf-18	35.6	-8.3	3.89	2.36	1.81	2.39	127.1	173.4
$M_{(44)}$-conf-19	22.7	-15.0	3.48	2.31	1.82	2.46	162.2	175.3
$M_{(44)}$-conf-20	37.5	-13.6	3.88	2.42	1.82	2.34	118.9	166.0
$M_{(44)}$-conf-21	18.1	-10.7	3.19	2.23	1.81	2.36	153.1	170.5
$M_{(44)}$-conf-22	30.6	-9.2	3.50	2.25	1.81	2.43	154.9	174.0
$M_{(44)}$-conf-23	27.0	-5.1	3.60	2.27	1.82	2.46	132.2	177.6
$M_{(44)}$-conf-24	36.0	-4.2	3.76	2.25	1.82	2.41	133.2	168.0
$M_{(44)}$-conf-25	29.8	-6.5	3.63	2.30	1.81	2.38	141.0	166.2
Avg. (Ā)	26.4 ± 7.2	-9.6 ± 5.4	3.70 ± 0.60	2.28 ± 0.05	1.81 ± 0.01	2.33 ± 0.08	133.9 ± 28.2	169.9 ± 6.0

Table S11. Selected geometric parameters for the conformations at the RS and TS for the Y44A methyl transfer reaction carried out by the mutated M3RA-Y44A. Energies are in kcal/mol. distances in Å and angles in degrees (°)

System	ΔE≠ ± S	$\Delta E_2°$ ± S	*C-N(RS) ± S*	*C-N(TS) ± S*	*S-C(RS) ± S*	*S-C(TS) ± S*	*S-C-N(RS) ± S*	*S-C-N(TS) ± S*
$M_{(44)}$-conf-1	20.4	-8.5	4.07	2.22	1.82	2.24	54.8	165.2
$M_{(44)}$-conf-2	11.1	-21.2	2.89	2.22	1.81	2.19	163.2	170.2
$M_{(44)}$-conf-3	13.3	-17.4	2.96	2.19	1.81	2.23	145.3	170.1
$M_{(44)}$-conf-4	12.3	-14.7	2.88	2.18	1.82	2.22	164.2	171.9
$M_{(44)}$-conf-5	17.5	-7.7	3.14	2.19	1.82	2.34	169.9	177.3
$M_{(44)}$-conf-6	14.2	-9.3	2.98	2.16	1.82	2.29	173.5	175.2
$M_{(44)}$-conf-7	10.1	-14.7	2.88	2.17	1.82	2.25	173.5	174.9
$M_{(44)}$-conf-8	9.6	-19.1	2.86	2.19	1.83	2.22	170.9	177.2
$M_{(44)}$-conf-9	11.6	-16.7	2.94	2.20	1.82	2.24	168.1	173.7
$M_{(44)}$-conf-10	11.8	-14.1	3.12	2.20	1.82	2.31	170.7	173.4
$M_{(44)}$-conf-11	14.8	-11.3	2.99	2.16	1.82	2.29	171.7	173.7
$M_{(44)}$-conf-12	15.9	-15.9	3.23	2.22	1.81	2.18	149.1	170.4
$M_{(44)}$-conf-13	17.4	-5.3	3.02	2.15	1.82	2.35	165.4	171.7
$M_{(44)}$-conf-14	13.4	-13.4	2.98	2.21	1.82	2.30	166.6	171.5
$M_{(44)}$-conf-15	15.5	-8.8	3.03	2.18	1.82	2.32	166.1	172.3
$M_{(44)}$-conf-16	11.0	-22.8	3.01	2.26	1.83	2.26	168.1	171.8
$M_{(44)}$-conf-17	15.5	-11.1	3.05	2.20	1.82	2.33	163.7	171.6
$M_{(44)}$-conf-18	13.5	-18.8	3.04	2.24	1.82	2.28	160.7	169.1
$M_{(44)}$-conf-19	14.6	-17.7	3.16	2.27	1.82	2.31	159.7	170.7
$M_{(44)}$-conf-20	11.7	-21.1	3.03	2.28	1.83	2.26	162.9	169.8
$M_{(44)}$-conf-21	12.5	-19.2	3.02	2.25	1.82	2.27	164.0	170.3
$M_{(44)}$-conf-22	14.4	-19.2	3.10	2.27	1.83	2.32	161.7	169.1
$M_{(44)}$-conf-23	31.2	-7.4	4.12	2.31	1.82	2.49	152.1	175.4
$M_{(44)}$-conf-24	19.4	-22.9	3.38	2.32	1.82	2.25	118.7	169.1
$M_{(44)}$-conf-25	35.2	-14.0	4.16	2.37	1.82	2.25	97.9	161.8
Avg. (Ā)	15.5 ± 6.0	-14.9 ± 5.1	3.16 ± 0.38	2.22 ± 0.06	1.82 ± 0.01	2.28 ± 0.06	155.3 ± 27.1	171.5 ± 3.4

Table S12. Selected geometric parameters for the conformations at the RS and TS for the Y128A methyl transfer reaction carried out by the mutated M3RA-Y128A. Energies are in kcal/mol. distances in Å and angles in degrees (°)

System	ΔE≠ ± S	$\Delta E_2°$ ± S	*C-N(RS) ± S*	*C-N(TS) ± S*	*S-C(RS) ± S*	*S-C(TS) ± S*	*S-C-N(RS) ± S*	*S-C-N(TS) ± S*
$M_{(128)}$-conf-1	36.3	0.7	4.45	2.3	1.82	2.27	58.4	171.2
$M_{(128)}$-conf-2	15.8	-18.8	4.89	2.28	1.82	2.21	41.5	177.4
$M_{(128)}$-conf-3	18.8	-18.8	3.13	2.2	1.81	2.2	101.9	169.1
$M_{(128)}$-conf-4	22.7	-9.4	3.77	2.18	1.82	2.21	72.1	170.5
$M_{(128)}$-conf-5	14.3	-4.3	2.97	2.24	1.82	2.27	174.2	175.2
$M_{(128)}$-conf-6	10.2	-16.6	2.99	2.25	1.83	2.27	150.9	176.0
$M_{(128)}$-conf-7	9.3	-21.7	2.97	2.32	1.83	2.26	169.7	172.2
$M_{(128)}$-conf-8	13.4	-26.4	3.13	2.27	1.81	2.27	131.4	171.3
$M_{(128)}$-conf-9	10.0	-17.9	2.92	2.24	1.82	2.24	157.3	170.6
$M_{(128)}$-conf-10	11.8	-20.6	2.92	2.21	1.81	2.28	164.0	172.6
$M_{(128)}$-conf-11	11.1	-14.9	3.04	2.19	1.8	2.18	128.5	166.8
$M_{(128)}$-conf-12	16.6	-17.7	3.17	2.29	1.82	2.4	167.4	171.9
$M_{(128)}$-conf-13	7.3	-11.8	3.06	2.23	1.8	2.15	136.6	171.3
$M_{(128)}$-conf-14	16.7	-23.8	3.32	2.3	1.82	2.34	137.3	166.2
$M_{(128)}$-conf-15	17.2	-18.6	3.22	2.23	1.81	2.29	115.6	166.7
$M_{(128)}$-conf-16	14.3	-11.4	3.07	2.27	1.82	2.34	159.7	173.4
$M_{(128)}$-conf-17	21.9	-42.5	3.41	2.27	1.82	2.42	141.9	173.0
$M_{(128)}$-conf-18	15.2	-15.1	3.11	2.25	1.81	2.31	133.8	171.3
$M_{(128)}$-conf-19	15.3	-15.1	3.18	2.26	1.81	2.32	130.9	177.8
$M_{(128)}$-conf-20	10.3	-14.6	3.04	2.22	1.81	2.25	133.4	173.5
$M_{(128)}$-conf-21	18.9	-19.6	3.27	2.3	1.82	2.38	172.9	172.0
$M_{(128)}$-conf-22	11.2	-16.7	3.11	2.34	1.82	2.29	152.9	173.5
$M_{(128)}$-conf-23	10.5	-25.0	2.94	2.27	1.83	2.28	169.6	173.9
$M_{(128)}$-conf-24	14.8	-20.4	3.02	2.24	1.83	2.33	172.8	176.3
$M_{(128)}$-conf-25	15.3	-12.5	3.06	2.27	1.82	2.33	160.4	168.2
Avg. (Ā)	15.2 ± 5.9	-17.3 ± 8.0	3.25 ± 0.47	2.26 ± 0.04	1.82 ± 0.01	2.28 ± 0.07	137.4 ± 36.0	171.8 ± 2.9

Table S13. Selected geometric parameters for the conformations at the RS and TS for the Y44A methyl transfer reaction carried out by the mutated M3RA-Y44A. Energies are in kcal/mol. distances in Å and angles in degrees (°)

System	ΔE≠ ± S	ΔE_2° ± S	*C-N(RS) ± S*	*C-N(TS) ± S*	*S-C(RS) ± S*	*S-C(TS) ± S*	*S-C-N(RS) ± S*	*S-C-N(TS) ± S*
$M_{(44)}$-conf-1	16.8	-8.6	5.01	2.14	1.83	2.24	37.4	174.3
$M_{(44)}$-conf-2	37.1	10.6	5.44	2.29	1.82	2.29	39.0	151.4
$M_{(44)}$-conf-3	31.8	5.2	5.47	2.15	1.81	2.29	43.0	163.5
$M_{(44)}$-conf-4	28.1	-2.6	5.53	2.25	1.82	2.28	38.9	166.2
$M_{(44)}$-conf-5	32.6	-0.9	5.59	2.28	1.82	2.26	38.7	164.8
$M_{(44)}$-conf-6	38.7	-1.6	5.23	2.26	1.82	2.42	75.9	175.0
$M_{(44)}$-conf-7	39.0	6.6	4.08	2.24	1.81	2.43	120.4	171.6
$M_{(44)}$-conf-8	21.7	-9.5	3.63	2.27	1.81	2.32	138.8	171.0
$M_{(44)}$-conf-9	25.9	-9.2	3.71	2.33	1.81	2.26	123.8	168.0
$M_{(44)}$-conf-10	22.9	-8.8	3.53	2.27	1.82	2.29	117.8	167.9
$M_{(44)}$-conf-11	22.3	-8.6	3.52	2.21	1.81	2.21	116.4	169.8
$M_{(44)}$-conf-12	21.5	-11.1	3.4	2.25	1.81	2.2	115.3	166.4
$M_{(44)}$-conf-13	23.5	-2.5	3.69	2.22	1.81	2.36	146.3	175.2
$M_{(44)}$-conf-14	27.8	-10.3	3.95	2.25	1.82	2.33	114.4	173.7
$M_{(44)}$-conf-15	49.0	-3.5	5.51	2.44	1.82	2.4	89.4	75.9
$M_{(44)}$-conf-16	47.2	0.6	4.72	2.47	1.82	2.32	112.6	162.4
$M_{(44)}$-conf-17	38.9	-1.5	4.4	2.26	1.81	2.48	122.1	171.8
$M_{(44)}$-conf-18	39.4	-3.9	4.42	2.28	1.82	2.43	104.8	164.4
$M_{(44)}$-conf-19	29.6	-12.1	3.69	2.22	1.81	2.34	114.1	170.2
$M_{(44)}$-conf-20	42.1	3.2	5.07	2.37	1.82	2.35	91.8	158.6
$M_{(44)}$-conf-21	30.6	-12.5	4.55	2.28	1.81	2.31	108.4	163.9
$M_{(44)}$-conf-22	31.4	-21.9	5.04	2.34	1.82	2.53	94.8	164.6
$M_{(44)}$-conf-23	61.1	-4.5	5.16	2.29	1.82	2.49	91.1	167.9
$M_{(44)}$-conf-24	60.9	-2.7	6.25	2.47	1.82	2.53	88.7	160.2
$M_{(44)}$-conf-25	44.0	-5.6	5.32	2.5	1.82	2.49	89.2	154.3
Avg. (Ā)	34.6 ± 11.7	-4.6 ± 7.0	4.64 ± 0.83	2.29 ± 0.09	1.82 ± 0.01	2.35 ± 0.10	94.9 ± 32.6	162.9 ± 19.1

REFERENCES

1. Miranda-Rojas, S.; Blanco-Esperguez, K.; Tuñón, I.; Kästner, J.; Mendizábal, F. Exploration of the Activation Mechanism of the Epigenetic Regulator MLL3: A QM/MM Study. *Biomol.* 2021, *11*.

2. Li, Y.; Han, J.; Zhang, Y.; Cao, F.; Liu, Z.; Li, S.; Wu, J.; Hu, C.; Wang, Y.; Shuai, J.; et al. Structural basis for activity regulation of MLL family methyltransferases. *Nature* **2016**, *530*, 447–452, doi:10.1038/nature16952.
